# Head‐down tilt lithotomy position and well‐leg compartment syndrome: An international survey of current practice

**DOI:** 10.1111/codi.70134

**Published:** 2025-06-07

**Authors:** Chukwuemeka C. Uzoma, Anthony I. Shepherd, Zoe L. Saynor, Jim S. Khan, Guglielmo Niccolò Piozzi, Rauand Duhoky, Christopher Askew, M. Mahir Ozmen, Thierry R. F. Middleton, Shamsul Masum, Maria Perissiou, HDTL‐WLCS Global Survey Collaborating Group, Aakansha Giri Goswami, Aamir Aziz, Abdolazeem Elnour, Abdourahmane Ndong, Abdul Fattah Bin Abdul Hamid, Abdullah Khan, Abdullahi Musa Kulfi, Adam Mylonakis, Ademola Adeyeye, Adriana Liceaga Fuentes, Agnė Miknevičiūtė, Ala Hassouneh, Alaa El‐Hussuna, Alazar Berhe Aregawi, Alberto Aiolfi, Alberto Arezzo, Alberto Ignacio Herrando, Alberto Vannelli, Alessia Malagnino, Alexander Julianov, Alexandros Kozadinos, Alexis Theodorou, Ali Cihat Yildirim, Ali Guner, Ali Malik, Ali Yasen Mohamedahmed, Alisina Bulut, Amedeo Antonelli, Amir Botros, Amro Ahmed Mureb, Andee Dzulkarnaen Zakaria, Andrea Divizia, Andrea Martina Guida, Andrea Romanzi, Andrew A. Gumbs, Andrew Craig Lynch, Andrew Day, Angelo Alessandro Marra, Angelo Parello, Anna Paspala, Anthony Lin, Antonio Castaldi, Antonio Luberto, Antonio Simone Laganà, Antonios Koutras, Arcangelo Picciariello, Argyrios Ioannidis, Aristeidis Papadopoulos, Arshad Rashid, Asad Ali Kerawala, Athanasios Marinis, Athanasios Pantelis, Athanasios Syllaios, Austin Acheson, Avanish Saklani, Ayandele Babajide Oladayo, Badri Kobalava, Beata Hemmelova, Beatrice Drago, Binyam Yohannes, Boby Sebastian, Carlo Ratto, Casoni Pattacini Gianmaria, Cem Emir Guldogan, Cezar Ionut Ciubotaru, Chigozie Innocent Onyeze, Christian Agbo Agbo, Christina Bali, Christina Fleming, Christina Kontopoulou, Christos Chouliaras, Cigdem Benlice, Cihad Tatar, Cihangir Akyol, Claudio Coco, Colin Peirce, Constantinos Nastos, Cristian Gallardo, Cristiano Huscher, Danette Wright, Daniel Wilby, Danielle Brogden, Daunia Verdi, David Merlini, David Watt, Davide Pertile, Dermot Burke, Despotidis Markos, Diego Sasia, Dimitrios Ntourakis, Dimitrios Papaconstantinou, Dimitrios Tsapralis, Dimitris P. Korkolis, Dimosthenis Chrysikos, Diogo Carrola Gomes, Douglas Bowley, Dragomir Dardanov, Ebbe Billmann Thorgersen, Egemen Ozdemir, Ekhlas Jabber Kadhim, Eleftherios Spartalis, Eleni Andriopoluou, Elisa Reitano, Elizabeth Li, Ellen Van Eetvelde, Emmanuel Akpo, Emre Gonullu, Emre Gunay, Enver Tekin, Ewen Harrison, Eyup Murat Yilmaz, Fabrizio Sammartano, Fang Yi Cheung, Farhanul Huda, Federica Di Marco, Feras AlJarad, Filippo Carannante, Firdaus Hayati, Floris Boudewijn Poelmann, Fodor Decebal, Frances Dixon, Francesco Bianco, Francesco Pata, Gabriela Arroyo Murillo, Gabriella Marchitelli, Gabrielle H. van van Ramshorst, Gaia Colletti, Ganendra Paramasvaran, Georgios Fragulidis, Georgios Rallis, Georgios Stravodimos, Gerald David, Giacomo Calini, Giacomo Carganico, Giampaolo Formisano, Gianluca Cassese, Gianluca Pellino, Giorgio Bogani, Giorgio Dalmonte, Giorgio Lisi, Giovanni Cestaro, Giulia Turri, Giuseppe Brisinda, Giuseppe Cucinella, Giuseppe Frazzetta, Gonzalo P. Martin‐Martin, Habeeb Olufemi Gbenga, Haluk Kerim Karakullukcu, Harald Krentel, Hasan Mukhtar, Hashim E. Elmansi Abdalla, Hemendra Kumar Mangal, Hossam Elfeki, Ian Daniels, Ibrahim Darwich, Ibrahim Umar Garzali, Ifeanyichukwu Kelvin Egbuchulem, Ilenia Merlini, İlgar Ismayilov, İlknur Turan, Ioannis Katsaros, Ioannis Virlos, Ionut Negoi, Irfan Ahmed, Jacopo Andreuccetti, James Glasbey, James Olivier, James Wheeler, Jamil Ahmed, Jan Cagaš, Jared Torkington, Jayesh Sagar, Jeremy Meyer, Jeremy Yuen‐Chun Teoh, Jin Jiun Mah, John Afam‐Osemene, Jonathan Lee, Joris P. Bulte, Joseph Mathew, Justin Davies, Kapil Sahnan, Kashish Malhotra, Kaushika Gunasekare, Kemal Erdinç Kamer, Khaled Rida, Kollaras C. Vasileios, Konstantinos Apostolou, Konstantinos Bouchagier, Konstantinos Kopanakis, Konstantinos Stratakis, Kosachenko Mikhail, Kris Jourand, Krunal Khobragade, Lasitha Bhagya Samarakoon, Lawal Bashir Oladimeji, Leandro Siragusa, Linardoutsos Dimitrios, Lopez‐Lopez Victor, Luca Domenico Bonomo, Luca Pio, Lucio Taglietti, Luigi Battaglia, Luigi Bonavina, Mah Muneer Khan, Malcolm A. West, Marco Cannistra, Marco Catarci, Marco Giacometti, Maria Chiara Sighinolfi, Maria Papadoliopoulou, Maria Sotiropoulou, Mario Trompetto, Marius Kryzauskas, Marius T. Paraoan, Mark A. Potter, Mark R. Brincat, Marta Spalluto, Martin Rutegård, Mauro Podda, Maximos Frountzas, Mehmet Ali Koç, Mehmet Ömer Özduman, Mejudin Kedir Abdella, Mert Guler, Michael Spartalis, Michail Vailas, Michel Adamina, Michele Ballabio, Mohamed A. Thaha, Mohamed Arif Hameed Sultan, Mohamed Ebrahim, Mohammad Faraz Khan, Mohammed Basheeruddin Inamdar, Mohammed Eid, Mohana Raj Thanapal, Mohd Syakir Mohd Azahar, Morini Andrea, Mostafa Shalaby, Muhammad Nur Syamim bin Che Johan, Muhammad Salman Shafique, Muhammad Shamim, Muhammad Umar Younis, Muhammer Ergenç, Mukoro Duke George, Murat Kalin, Mustafa Yener Uzunoglu, Naciye Cigdem Arslan, Narimantas Evaldas Samalavicius, Nathan Curtis, Navin Kumar, Nguyen Thanh Sang, Nicholas Wong, Nicola Cinardi, Nicola de’Angelis, Nicolas Flamey, Nicolò Tamini, Nikolaos Chatzizacharias, Nikolaos Koliakos, Nikolaos Machairas, Nikolaos V. Michalopoulos, Nnaemeka Eli, Nurhilal Kızıltoprak, Nuri Okkabaz, Nurudeen Akinbami, Okechukwu Hyginus Ekwunife, Omer Faruk Ozkan, Omer Yalkin, Omorodion Irowa, Orçun Yalav, Orestis Ioannidis, Pamela Milito, Panteleimon Vassiliu, Paolo Delrio, Pascal Herzog, Patricia Tejedor, Paul Okeny, Peter Ihnát, Peter Ikponmwosa Agbonrofo, Petr Vlček, Philip H. Pucher, Pietro Fransvea, Prashant Naik, Prem Thambi, Promise Wichendu, Rachel McKinney, Rafael Garatea Grau, Rafael Sanchez Salas, Raffaele Galleano, Raimundo Izquierdo, Rajeev Peravali, Rany Aoun, Ravi Aggarwal, Rebecca Reid, Reinaldo Isaacs, Renan Carlo Colombari Monteiro, Renato Gomes Campanati, Rikesh Patel, Roberto Sampietro, Rogier Crolla, Romulo R. Cabantac, Ronald Mbiine, Rubén Domínguez Azuaga, Saburi Oyewale, Sajith Pankajavihar Sasi, Salih Müjdat Balkan, Salomone Di Saverio, Samuel Stefan, Santoro Giulio Aniello, See Boon Keong, Semra Demirli Atici, Sentilnathan Subramaniam, Seon Hahn Kim, Sergey Efetov, Sergio M. Navarro, Sevcan Arzu Arinkan, Sezai Leventoglu, Shafaque Shaikh, Simon Middleton, Simone Manfredelli, Somprakas Basu, Spyridon Christodoulou, Spyridon Dritsas, Stefano D’Ugo, Stoica Bogdan, Stylianos Kapiris, Summi Karn, Sylvia Krivan, Sztipits Tamás, Tahsin Colak, Tevfik Kıvılcım Uprak, Thalia Petropoulou, Theodoros Sidiropoulos, Tigabu Daniel Ayase, Tijmen Koëter, Tommaso Dominioni, Tommaso Fontana, Tommaso Violante, Usman Gwaram, Uzochi Ebochue, Valentin Calu, Venkatesh Munikrishnan, Vikas Sud, Vincenzo Vigorita, Vittoria Bellato, Vittorio Bresadola, Vusal Aliyev, Wasim Dar, Zafar Ahmed Khan, Zafer Şenol, Zainab Obaid Jaddoa, Zampitis Nikolaos, Zhang Yankai, Zoe Garoufalia, Zubair ud Din, Zygomalas Apollon, Εlissavet Anestiadou, Harish Neelamraju Lakshmi, Tomara Nefeli‐Kaiti, Mehmet Ayhan Kuzu, Paola De Nardi, Olivier Iryivuze, Sudhir Dhaygude, Nadzlee Harith Bin Paisol, Sidra Rauf, Denis Tsepov, Mahmudul Hasan, Justin Alberts, Akshay Bavikatte Prasannakumar, Dorottya Turu, Ahmed Adam, Cumhur Yesildal, Hazim Eltyeb, Pablo Baeza‐Ibáñez, Mohd Azharuddhin, Ahmad Amhaimed, Claudia Alejandra Antón Velez, Ouazzani Et‐Tayab, William Speake, Emre Furkan Kirkan, Megha Mishra, Ioannis Paraskevopoulos, Biagio Picardi, Braulio Francisco Reyes Méndez, Victor Bako, Riza Deryol, Henok Teshome Ayele

**Affiliations:** ^1^ Clinical Health and Rehabilitation Team, School of Psychology, Sport and Health Sciences University of Portsmouth Portsmouth UK; ^2^ Department of Faculty Surgery RUDN University Moscow Russia; ^3^ School of Health Sciences, Faculty of Environmental and Life Sciences University of Southampton Southampton UK; ^4^ Department of Colorectal Surgery Portsmouth Hospitals University NHS Trust Portsmouth UK; ^5^ Faculty of Science and Health University of Portsmouth Portsmouth UK; ^6^ School of Computing, Faculty of Technology University of Portsmouth Portsmouth UK; ^7^ VasoActive Research Group, School of Health University of the Sunshine Coast Sunshine Coast Queensland Australia; ^8^ Mahir Ozmen Clinic (MOC) Ankara Turkey; ^9^ Sapienza University of Rome, Medical School Rome Italy; ^10^ School of Electrical and Mechanical Engineering University of Portsmouth Portsmouth UK

**Keywords:** compartment syndrome, global survey, Lloyd‐Davies, perioperative practices, prevention and management, robotic surgery

## Abstract

**Aim:**

Well‐leg compartment syndrome (WLCS) is a serious complication of prolonged surgery in the head‐down tilt lithotomy (HDTL) position associated with increased postoperative morbidity and mortality. However, there is a lack of awareness and clinical guidance regarding prevention of WLCS. The aim of this study was to assess current HDTL‐related practices and occurrence of WLCS among a global cohort of clinicians.

**Method:**

An international online survey of clinicians was conducted between July and December 2023. Data analysis involved descriptive statistics, machine learning techniques and qualitative content analysis.

**Results:**

A total of 595 clinicians from 71 countries and 14 specialities participated. Most (98%) reported routine use of HDTL, 27% of whom did not implement any preventive strategies. ‘Leg rest’ was the most reported preventive measure (41%), commonly initiated after 2 or 3 h of HDTL (79%), for 10–15 min (56%). Overall, 170 cases of WLCS were reported by 21% of respondents. The majority reported unilateral WLCS (81%) following a laparoscopic procedure (63%) performed in HDTL (64%). Only 28% of respondents discussed WLCS during consent for operations in HDTL. Machine learning identified ‘duration of uninterrupted HDTL’ as a positive predictor of the occurrence of WLCS (*p* < 0.001). Content analysis demonstrated that clinician perspectives and practices regarding WLCS are significantly influenced by personal experience, mostly due to a poor evidence base and lack of standardized institutional policies.

**Conclusion:**

Perioperative practices during procedures in HDTL vary substantially, and are primarily informed by clinician experience and preferences. There is a need for evidence‐based consensus on best practices to enhance safety during procedures in HDTL.

**What does this paper add to the literature?**

This paper provides the first international survey to assess practices and clinician perspectives regarding head‐down tilt lithotomy (HDTL) and well‐leg compartment syndrome (WLCS). It highlights substantial variability in perioperative practices, identifies key predictors of WLCS and emphasizes the need for evidence‐based guidelines to enhance patient safety during procedures in HDTL.

## INTRODUCTION

Minimally invasive surgery (MIS) has revolutionized surgical practice through the use of smaller incisions that enable more efficient operations [1, 2], with improved morbidity [1, 2, 3, 4, 5, 6, 7, 8] and mortality [8, 9]. MIS has become the standard of care for many abdominopelvic conditions [5, 10]. Despite its benefits, abdominopelvic MIS often requires prolonged, nonphysiological surgical positions, such as the head‐down tilt lithotomy (HDTL) position [11]. Commonly employed during colorectal, gynaecological and urological MIS [12, 13], HDTL leverages gravity to achieve visceral retraction and exposure, facilitating simultaneous access to the pelvis and perineum [11, 14].

Prolonged HDTL, however, is associated with a unique form of compartment syndrome, well‐leg compartment syndrome (WLCS) [15, 16, 17, 18, 19, 20, 21, 22, 23, 24, 25]. WLCS manifests as acute intracompartmental hypertension, leading to tissue ischaemia in a previously uninjured (‘well’) leg [25, 26], and is primarily observed after extended surgery (>4 h) [23, 25, 27, 28, 29] in the lithotomy [17, 30, 31, 32] or HDTL [12, 20, 25, 33] positions. Delayed recognition and management of WLCS often leads to significant postoperative morbidity [25, 30, 34, 35], permanent disability [23, 30, 31] or death [25, 36, 37]. Although the reported incidence of WLCS ranges from 0.03% to 0.80% [12, 23, 27, 38, 39], this is probably an underestimate of the true incidence due to low clinical awareness [40, 41, 42] and underreporting [13, 23, 25]. Furthermore, the increasing global adoption of MIS in abdominopelvic surgery is likely to result in a concurrent rise in incidence of WLCS [25, 43], particularly during the early learning curve period [27].

However, owing to the rarity of WLCS and a limited evidence base [25] there is a poor awareness of the condition among clinicians and a lack of standardized guidance for its prevention [13, 17, 25, 39, 44]. This study aimed to explore current HDTL‐related practices and assess the occurrence of WLCS among a global cohort of clinicians who routinely use HDTL.

## METHOD

### Study design

This cross‐sectional online survey adopted a mixed‐methods embedded design (Figure S1), with qualitative findings supplementing the primary quantitative data [45]. Ethics approval was granted by the Faculty of Science and Health Ethics Committee, University of Portsmouth (SHFEC 2023‐057). The study was preregistered on the Open Science Framework (OSF, www.osf.io/r82cq) and the dataset is available via the OSF registry (https://doi.org/10.17605/OSF.IO/YR7KD). The survey followed the Checklist for Reporting Of Survey Studies (CROSS) Checklist [46] (Table S1).

### Participants

Convenience sampling was used to recruit clinicians who routinely employ HDTL in their practice. The survey was open to consultants/attendings, clinical fellows or career‐grade specialist trainees in general surgery, colorectal surgery, lower and upper gastrointestinal surgery, gynaecology and urology (Table 1).

**TABLE 1:** Characteristics of survey participants (*N* = 595).

	*n*.	%
Age group (years)		
20–29	24	4.0
30–39	257	43.2
40–49	191	32.1
50–59	95	16.0
≥60	25	4.2
I prefer not to say	3	0.5
Gender		
Female	90	15.1
Male	480	80.7
I prefer not to say	25	4.2
Surgical speciality^a^		
Colorectal surgery	391	65.7
General surgery	376	63.2
Lower GI	148	24.9
Upper GI	102	17.1
Obstetrics and gynaecology	26	4.4
Urology	19	3.2
Other^b^	24	4.0
Global regions		
Europe	442	74.3
Asia	70	11.8
Africa	46	7.7
South America	20	3.4
North America	11	1.8
Oceania	6	1.0
MIS experience		
No	9	1.5
Yes	586	98.5
Length of MIS experience		
<5 years	161	27.1
5–10 years	183	30.8
>10 years	242	40.7
Routine use of HDTL in practice		
No	12	2.0
Yes	583	98.0
Ever encountered WLCS in your practice		
No	473	79.5
Yes	122	20.5

Abbreviations: GI, gastrointestinal; HDTL, head‐down tilt lithotomy; MIS, minimally invasive surgery; WLCS, well‐leg compartment syndrome.

^a^ Multi‐answer question—the percentage of respondents who selected each answer option (e.g. 100% would represent that all this question's respondents chose that option).

^b^ Other specialities: paediatric surgery (*n* = 6), surgical oncology (*n* = 4), peritoneal malignancy (*n* = 3), hepatobiliary surgery (*n* = 2), trauma and orthopaedic surgery(*n* = 2), vascular surgery (*n* = 1), transplant surgery (*n* = 1), bariatric and metabolic surgery (*n* = 1), abdominal wall surgery (*n* = 1), anaesthesia (*n* = 1).

Social media platforms (X™ and LinkedIn™), relevant clinical associations [Association of Laparoscopic Surgeons of Great Britain and Ireland (ALSGBI), Turkish Society of Colon and Rectal Surgery, Association of Coloproctology of Great Britain and Ireland, Indian Association of Surgical Oncology, European Society of Coloproctology] and scientific conferences (ALSGBI) facilitated survey distribution.

Participants provided informed consent prior to completing the anonymous survey and could withdraw at any time by not submitting their responses. A link on the final page invited participants to join the collaborating group (Appendix S1).

### Survey tool

A 34‐item survey (Appendix S1) was developed by a multidisciplinary team of clinicians and researchers, after reviewing relevant literature [12, 13, 17, 23, 27, 47]. Piloted by independent clinicians for clarity and functionality, it was revised based on feedback. This survey was administered in English via Online Surveys (JISC, Bristol, UK) from July to December 2023. Questions were structured into three main categories: (1) participant demographics; (2) HDTL perioperative practices and perceived WLCS risk factors; (3) WLCS incidence, surgical characteristics of reported WLCS cases and management strategies employed. The survey included single‐choice, multiple‐choice and open‐ended questions for respondents to elaborate on their answers.

### Data analysis

Response data were exported into Microsoft Excel 2019, IBM SPSS version 27 and Google Colaboratory (Python version 3.10) for analysis. Descriptive statistics characterized sociodemographics, HDTL practices, WLCS occurrences and preventive strategies for WLCS. Data were reported at a respondent level unless otherwise stated. Machine learning (ML) techniques from the ‘Scikit‐learn (sklearn)’ library [48] were employed to identify potential predictors of WLCS occurrence. Algorithms utilized included ordinary least squares [49], recursive feature elimination [50] and least absolute shrinkage and selection operator with cross‐validation (LassoCV) [51], trained on predictor variables (*x*; different survey questions) and the target variable (*y*; ever encountered WLCS in practice).

Open‐ended responses were analysed using inductive content analysis by first open‐coding the textual responses to generate subcategories and then grouping related subcategories into main categories [52].

## RESULTS

### Baseline characteristics

A total of 595 clinicians (Table S2) from 71 countries across six continents participated (Table 1, Figure S2). The most represented countries included the United Kingdom (*n* = 148, 24.9%), Italy (*n* = 95, 16.0%), Greece (*n* = 65, 11.4%) and Turkey (*n* = 52; 8.7%). Full country characteristics are shown in Table S3.

### HDTL clinical practice

**FIGURE 1:**
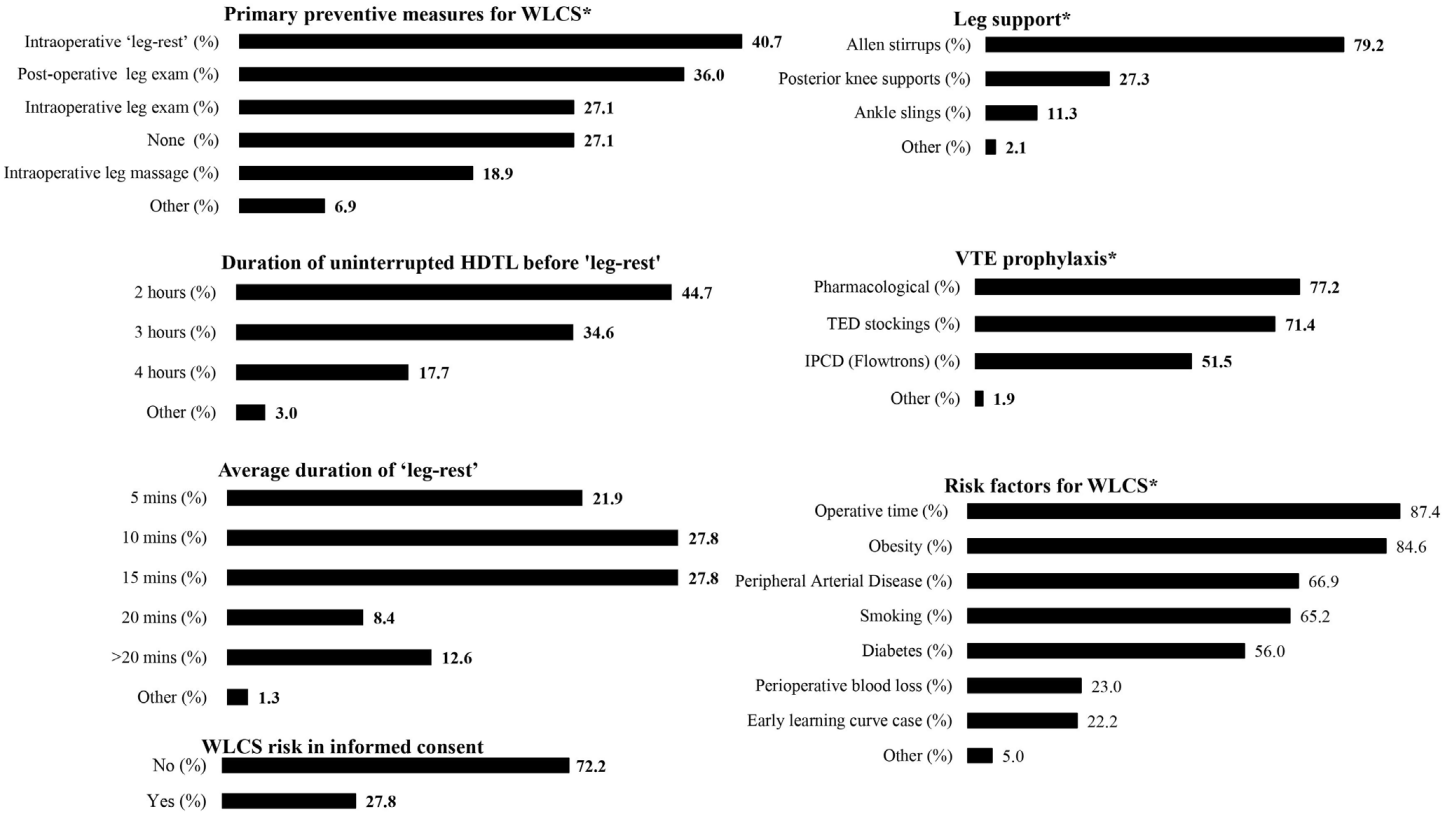
Current clinical practices and perceived risk factors for well‐leg compartment syndrome (WLCS). Data are presented as a proportion of the total number of responses for each question. HDTL, head‐down tilt lithotomy; IPCD, intermittent pneumatic compression device; TED, thromboembolic deterrent; VTE, venous thromboembolism. *Multi‐answer question—respondents had the option of more than answer.

Most respondents (*n* = 583, 98%) reported routine use of HDTL. Frequencies of reported clinical practices and perceived WLCS risk factors are presented in Figure 1. Notably, 158 respondents (27.1%) did not routinely implement preventative strategies for neuromuscular complications during procedures in HDTL. Intraoperative ‘leg‐rest’ protocols (return to supine or lowering legs below the heart level) were reported by 237 respondents (40.7%). These are typically implemented after 2 h (*n* = 106, 44.7%) or 3 h (*n* = 82, 34.6%) of uninterrupted HDTL, with rest durations of 10 min (*n* = 66, 27.8%) or 15 min (*n* = 66, 27.8%). Reported ‘leg‐rest’ protocols are presented in Table S4. Furthermore, 162 respondents (27.8%) confirmed discussion of WLCS risk during consent for operations requiring HDTL. Operating time (*n* = 520, 87.4%) and obesity (*n* = 502, 84.6%) were the key perceived risk factors, whereas ‘early learning curve cases’ was the least recognized (*n* = 132, 22.2%; Figure 1).

### Reported incidence of WLCS

**FIGURE 2:**
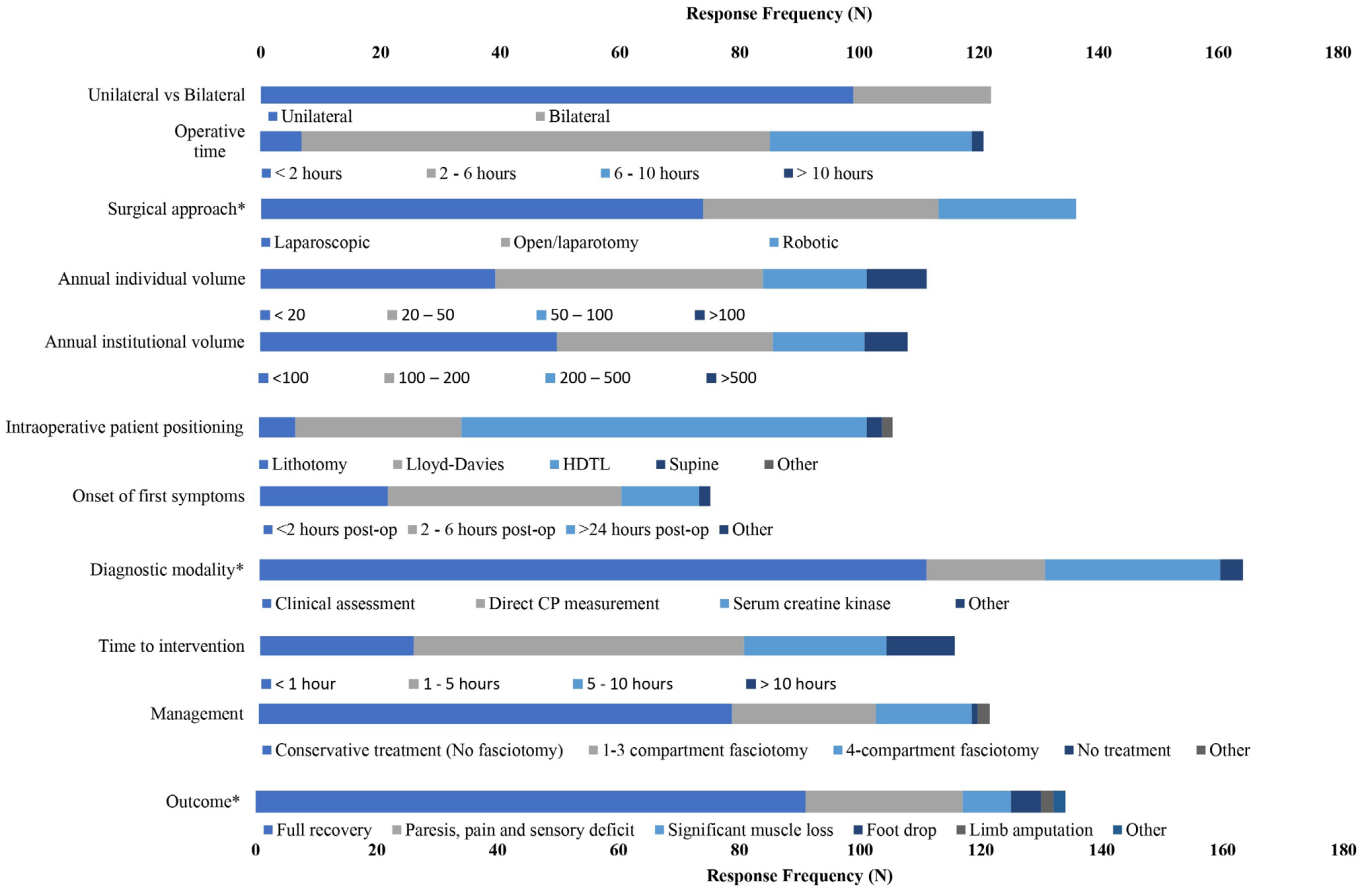
Characteristics of the reported cases of well‐leg compartment syndrome (WLCS). CP, compartment pressure; HDTL, head‐down tilt lithotomy. *Multi‐answer question—respondents had the option of more than answer.

Overall, 170 cases of WLCS were reported by 122 participants (20.5%; Figures 2 and S3). Among these surgeons, most reported lower annual individual (<50 surgeries, 75.4%) and institutional (<200 surgeries, 77.9%) operative volumes. Many had encountered only one case of WLCS (*n* = 85, 69.7%), with unilateral WLCS being more prevalent (*n* = 99, 81.1%). Most respondents reported WLCS following laparoscopic procedures (*n* = 77, 63.1%) performed in HDTL (*n* = 78, 64.2%), typically lasting 2–6 h (*n* = 79, 64.8%). Table S5 shows the surgical procedures preceding reported WLCS cases. Symptoms usually presented 2–24 h postoperatively (*n* = 83, 68.0%), and clinical assessment was the most commonly reported diagnostic modality (*n* = 77, 63.1%). Only 77 respondents (22.1%) reported intervention within 1 h of diagnosis, with delays of up to 24 h noted in three cases, one of which resulted in below‐the‐knee amputation. Conservative treatment and fasciotomy were reported by 79 (64.8%) and 40 (32.8%) respondents, respectively. Outcomes included full recovery (*n* = 91, 74.6%), foot drop (*n* = 5, 4.1%) and lower limb amputation (*n* = 1, 0.8%). One case reportedly resulted in successful litigation. No deaths were reported.

### Predictive variables of WLCS occurrence

**FIGURE 3:**
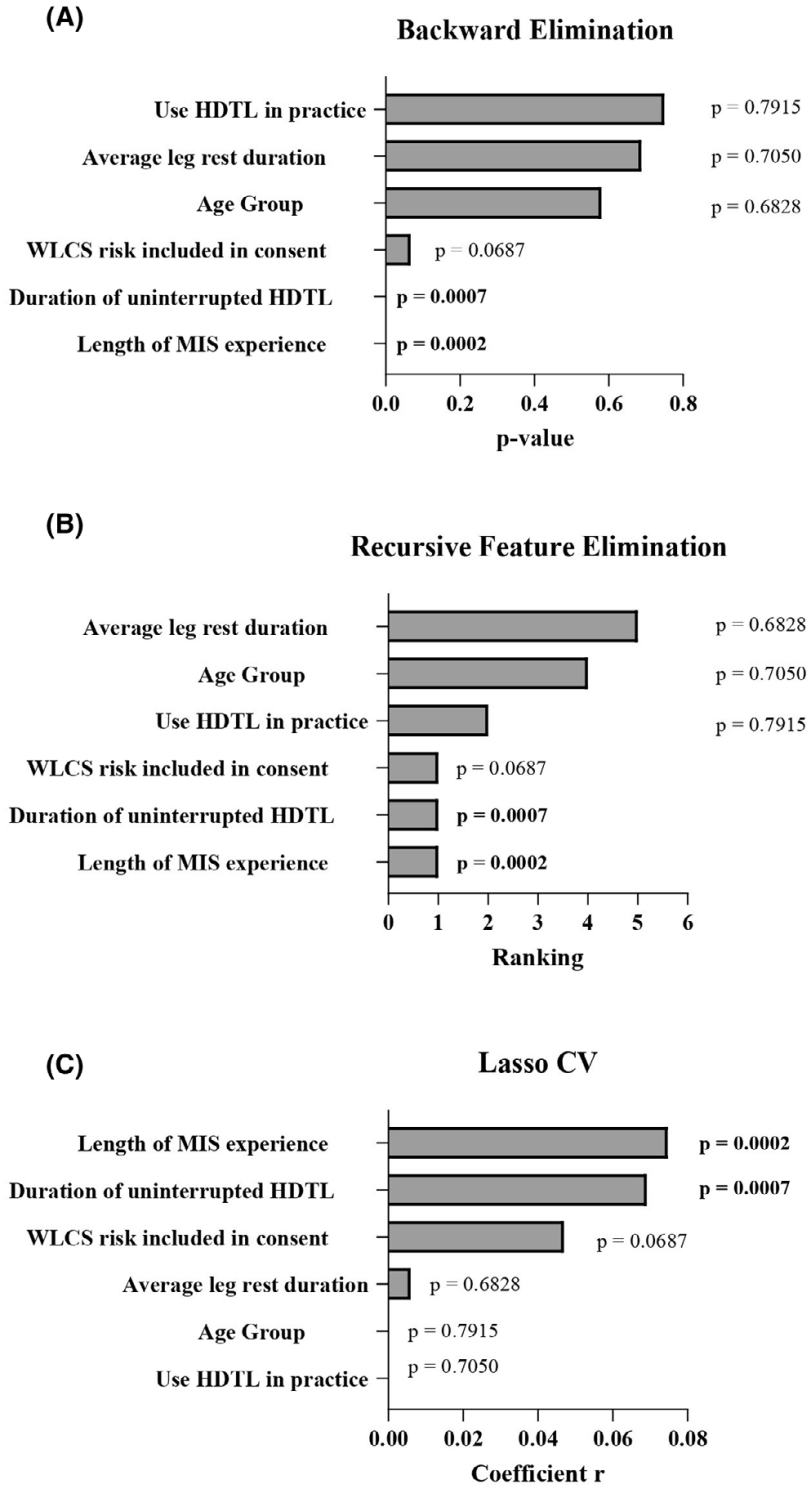
Predictive variables for the occurrence of well‐leg compartment syndrome (WLCS). (A) Ordinary least squares (OLS) [49] regression performs backward elimination by iteratively fitting OLS models and excluding the variable with the highest *p*‐value > 0.05 until all remaining variables have *p*‐values < 0.05. (B) Recursive feature elimination (RFE) [50] employs a linear regression model to select relevant variables, ranking them according to importance. Variables assigned a rank of 1 are considered the most important, with *p*‐values indicating their statistical significance. (C) Least absolute shrinkage and selection operator with cross‐validation (LassoCV) [51] identifies important variables via regularization and selection of the best parameter through cross‐validation. Some coefficients are set to zero, with nonzero coefficients corresponding to the important variables. HDTL, head‐down tilt lithotomy; MIS, minimally invasive surgery.

All ML algorithms, employing different computational processes, identified ‘length of MIS experience’ and ‘duration of uninterrupted HDTL’ as statistically significant predictors (*p* < 0.001) of the occurrence of WLCS in the survey cohort (Figure 3A–C). The distribution of reported WLCS occurrence across different levels of these two selected variables further illustrates patterns of association (Figure S4A,B).

### Qualitative analysis

Following an inductive content analysis, four main categories were generated from the qualitative responses provided by participants (Table S6).

#### ‘Leg rest’/patient repositioning as a preventive measure

Qualitative responses demonstrated substantial variations in intraoperative ‘leg‐rest’ practices, primarily underpinned by a lack of formal and standardized policies. Reported practices included ad hoc surgeon‐led approaches, stage‐guided strategies and integrated protocols incorporating leg rests with other measures. Regardless of approach, some respondents highlighted the importance of a team‐led approach rather than one solely dependent on the surgeon.

#### WLCS experience impacts perception and practice

Many participants emphasized the rarity of WLCS, supporting a clear association between experiencing WLCS and attitudes towards its prevention. Clinicians who had encountered WLCS were more likely to advocate for preventive protocols, while those with no such experience often perceived it as a low‐probability event not warranting significant attention.

#### Further research

Several respondents highlighted an inadequate awareness of WLCS among clinicians and the lack of a reliable evidence base as well as effectively communicated standardized strategies to mitigate WLCS. Additionally, many respondents indicated interest in actively contributing to collaborative research on this subject, with some proposing specific research questions to guide future investigations.

#### Considerations for practice

Free‐text responses also provided prospective considerations for improving clinical practice. Multiple surgeons highlighted the importance of incorporating WLCS risk into informed consent discussions for HDTL procedures. Implementation of pre‐ and postoperative preventive measures and exploring alternative surgical positions were proposed as viable strategies to minimize the risk of WLCS.

## DISCUSSION

This is the first international, multispeciality survey to evaluate clinical practices and clinician perspectives regarding HDTL and WLCS. The survey findings reveal substantial variation in current perioperative HDTL practices, with some clinicians not implementing any preventive measures. This study highlights that previous experience of WLCS strongly influences clinicians’ attitudes towards this complication, ultimately informing their clinical practice. Additionally, clinicians who routinely maintain longer periods of uninterrupted HDTL are more likely to encounter WLCS.

Survey responses demonstrate a lack of standardization towards prevention of WLCS among clinicians. While over a quarter (27.1%) of respondents do not utilize any preventive measures, there is considerable variability in practice among those who do. Some clinicians reportedly employ only one measure (‘leg rest’, leg examinations or leg massages), whereas others combine multiple approaches. ‘Leg‐rest’ protocols varied among respondents who employ this strategy. The majority (79.3%) reportedly reposition the legs after 2 or 3 h of HDTL, aligning with the UK and Ireland multidisciplinary guidelines published in 2019 [25] that recommend limiting uninterrupted leg elevation to 4 h. However, free‐text responses suggest that many clinicians are unaware of existing clinical guidance and/or do not have formal local institutional policies to inform practice. Hence, many have resorted to anecdotal strategies, leading to what seems to be an overall unstandardized approach to prevention of WLCS in current surgical practice regionally and globally. Respondents also highlighted a poor evidence base and insufficient clinical awareness as key contributors to variability in practice, emphasizing the need for further research. Considering the above, the authors assert that effective dissemination of evidence‐based consensus on best practices will be instrumental in standardizing WLCS prevention.

In this survey, one in five respondents (20.5%) reported at least one case of WLCS, with a total of 170 cases reported. This possibly lends credibility to the notion that WLCS is more prevalent than previously assumed [13, 23, 25]. WLCS was not reported by respondents who had no MIS experience or by those who do not use HDTL, underscoring the increased risk for WLCS associated with these techniques. The growing global adoption of abdominopelvic MIS, characterized by steeper head‐down tilts (≥15°) [16], longer operating times [16, 53] and impaired venous return due to pneumoperitoneum [28] may result in a rise in WLCS incidence. In the surveyed cohort, 43% of the respondents who utilize a 4‐h repositioning protocol had encountered WLCS compared with 20% and 30% of those who use 2‐ or 3‐h protocols, respectively. This finding suggests that ‘leg rests’ after every 2–3 h of HDTL may be associated with a lower incidence of WLCS. Moreover, ML analysis identified ‘duration of uninterrupted HDTL’ as a significant predictor for WLCS occurrence. Consequently, it may be prudent to revise relevant guidelines [25] and institutional policies that are currently predicated on a ‘safe’ 4‐h window of uninterrupted HDTL. Future research is needed to robustly evaluate these findings and establish optimal repositioning protocols.

Current literature suggests that many surgeons and institutions adopt a reactive approach to prevention, typically taking measures only after encountering WLCS [17, 28, 39, 42, 43]. Indeed, among survey respondents who have encountered WLCS, only 9% do not use any prevention protocols. While reactive measures are valuable, a proactive approach would be likely to enhance safety during abdominopelvic procedures involving HDTL. Qualitative data from this survey show that clinicians who have experienced WLCS strongly support the implementation of preventive protocols, which highlights the impact of personal experience on clinicians' attitudes and practices.

Despite the serious consequences associated with WLCS, many respondents (72%) do not routinely inform people about the risk of this complication before procedures requiring HDTL. Notably, prior WLCS experience did not substantially affect this practice, as only 36% of respondents who had encountered the complication reportedly include WLCS in consent discussions. This phenomenon may reflect the ongoing challenge clinicians face in determining which rare complications merit discussion during consent. The UK–Ireland guidelines [25] suggest that WLCS is insufficiently common to mandate routine discussion during consent, emphasizing the need to consider preferences and circumstances. Conversely*,* other guidance documents from the UK General Medical Council [54], the International Federation of Gynaecology and Obstetrics [55] and the Royal College of Surgeons of England [56] stress the importance of mentioning ‘material risks’ during consent, including any serious risks and/or those that a reasonable person would deem significant, regardless of their likelihood of occurring. This inconsistency possibly contributes to variations in consent practices across healthcare systems and may leave patients inadequately informed about the risks associated with their surgery. Given the ethical and legal implications of this situation, the authors propose developing an expert‐driven consensus on best practices that should, among other things, recommend routine discussion of the risk of WLCS during preoperative consultations for procedures in HDTL.

### Future directions

Standardization of clinical approaches to HDTL and WLCS in abdominopelvic surgery should be established through international guidelines informed by expert consensus and a comprehensive review of existing evidence. Clear and effective communication of these recommendations will promote consistency in practice across diverse healthcare settings. Prospective studies are needed to strengthen the evidence base, evaluate the safety and efficacy of current practices and identify areas for improvement. Incorporating teaching modules on WLCS into training programmes for surgeons and allied healthcare professionals as well as developing centralized national registries for WLCS cases will raise awareness and help reduce its incidence. These steps will foster evidence‐based practices and ultimately improve surgical care.

### Study limitations

Several limitations should be acknowledged when interpreting the findings of this study. First, convenience sampling may have introduced some selection bias. However, it is important to note that the survey responses did not reveal any characteristics that would significantly distinguish the respondents from the general surgical population. Secondly, the exact response rate could not be determined, making it difficult to assess the impact of nonresponse bias. This limitation is inherent in open surveys and is documented in the literature [57, 58]. Thirdly, the uneven distribution of participants across geographical regions (with 74% from Europe) and specialities (64% from colorectal/general surgery) may limit the generalizability of the findings. Nevertheless, the broad representation from 71 countries and 14 specialities offers a diverse perspective of clinical practices and attitudes. Lastly, the survey data on previous WLCS cases relied on respondents' memory, which introduces the potential for recall bias. Additionally, a more detailed subgroup analysis to examine the impact of specific variables on incidence of WLCS was not feasible due to missing data.

## CONCLUSION

Perioperative practices during HDTL procedures show considerable variability, influenced by clinicians' prior experience of WLCS and personal preferences, rather than standardized guidelines. This underscores the urgent need for future research to develop, validate and effectively disseminate evidence‐based protocols. Establishing these guidelines globally will be essential for ensuring consistent, high‐quality care in abdominopelvic surgery.

## AUTHOR CONTRIBUTIONS

**Chukwuemeka C. Uzoma:** Conceptualization; methodology; investigation; software; data curation; project administration; formal analysis; validation; writing – original draft; writing – review and editing; visualization. **Anthony I. Shepherd:** Conceptualization; methodology; supervision; writing – review and editing; funding acquisition; project administration; validation. **Zoe L. Saynor:** Conceptualization; methodology; supervision; writing – review and editing; data curation. **Jim S. Khan:** Conceptualization; methodology; supervision; writing – review and editing; data curation; validation; resources. **Guglielmo Niccolò Piozzi:** Data curation; writing – review and editing; resources; supervision; validation. **Rauand Duhoky:** Software; data curation; supervision; writing – review and editing; validation. **Christopher Askew:** Methodology; data curation; supervision; writing – review and editing; validation. **M. Mahir Ozmen:** Data curation; writing – review and editing. **Thierry R. F. Middleton:** Supervision; validation; writing – review and editing; formal analysis. **Shamsul Masum:** Software; validation; writing – review and editing; resources; supervision; formal analysis. **Maria Perissiou:** Conceptualization; methodology; software; data curation; supervision; formal analysis; validation; funding acquisition; writing – original draft; writing – review and editing; project administration; resources; visualization.

## FUNDING INFORMATION

This project was funded by the Faculty of Science and Health, University of Portsmouth.

## CONFLICT OF INTEREST STATEMENT

The authors declare no conflict of interest.

## ETHICS STATEMENT

Approval for this study was provided by the Faculty of Science and Health Ethics Committee, University of Portsmouth (SHFEC 2023‐057).

## Supporting information


Data S1.

## Data Availability

The dataset utilised in the preparation of this manuscript is available on the Open Science Framework (10.17605/OSF.IO/YR7KD).
