# Supplementary material for: Head‐down tilt lithotomy position and well‐leg compartment syndrome: An international survey of current practice
Source: Colorectal Dis. 2025 Jun 7;27(6):e70134. doi: 10.1111/codi.70134 (PMC12144681; doi:10.1111/codi.70134)
Supplement: Supplementary file 1 — Data S1. [file CODI-27-0-s001.docx]

# **SUPPLEMENTARY MATERIAL**

**Head-Down Tilt Lithotomy Position and Well-Leg Compartment Syndrome: An International Survey of Current Practice**

**Authors and institutions**

Chukwuemeka C. Uzoma^1,2^, Anthony I. Shepherd^1^, Zoe L. Saynor^3^, Jim S. Khan^4,5^, Guglielmo Niccolò Piozzi^4^, Rauand Duhoky^4,6^, Christopher Askew^7^, M Mahir Ozmen^8,9^, Thierry R. F. Middleton^1^, Shamsul Masum^10^, Maria Perissiou^1*^, HDTL-WLCS Global Survey Collaborating Group *(a full list of PubMed-citable collaborators is provided in Appendix S2)*

^1^Clinical Health and Rehabilitation Team, School of Psychology, Sport and Health Sciences, University of Portsmouth, Portsmouth, UK

^2^Department of Faculty Surgery, RUDN University, Moscow, Russia

^3^School of Health Sciences, Faculty of Environmental and Life Sciences, University of Southampton, Southampton, UK

^4^Department of Colorectal Surgery, Portsmouth Hospitals University NHS Trust, Portsmouth, UK

^5^Faculty of Science and Health, University of Portsmouth, Portsmouth, UK

^6^School of Computing, Faculty of Technology, University of Portsmouth, Portsmouth, UK

^7^VasoActive Research Group, School of Health, University of the Sunshine Coast, Australia

^8^Mahir Ozmen Clinic (MOC), Cukurambar, Ankara, Turkey

^9^Sapienza University of Rome, Medical School, Rome, Italy

^10^School of Electrical and Mechanical Engineering, University of Portsmouth, Portsmouth, UK

**Corresponding author.** Dr Maria Perissiou, Clinical Health and Rehabilitation Team, School of Psychology, Sport and Health Sciences, Faculty of Science and Health, University of Portsmouth, Portsmouth, PO1 2ER, Hampshire, United Kingdom. Email: [maria.perissiou@port.ac.uk](mailto:chukwuemeka.uzoma@port.ac.uk) **ORCID ID**: 0000-0002-3974-2250 **Twitter**: @maria_xper

**Supplementary Materials Index**

| **Supplementary Figures** |  |
| --- | --- |
| Figure S1. The embedded mixed-method design | *pag. 2* |
| Figure S2. Geographical distribution of survey respondents by country | *pag. 2* |
| Figure S3. Geographical distribution of the number of well-leg compartment syndrome cases reported by country | *pag. 3* |
| Figure S4. Distribution of the reported occurrence of well-leg compartment syndrome across ‘Duration of uninterrupted HDTL’ and ‘Length of MIS Experience’ | *pag. 3* |
| **Supplementary Tables** |  |
| Table S1. Checklist for Reporting Of Survey Studies (CROSS) Checklist | *pag. 4* |
| Table S2. Respondent progress | *pag. 7* |
| Table S3. Characteristics of countries | *pag. 8* |
| Table S4. Respondent-reported specific leg-rest protocols | *pag. 11* |
| Table S5. Surgical procedures that preceded the reported cases of well-leg compartment syndrome cases | *pag. 12* |
| Table S6. Representative quotes from free-text survey responses | *pag. 15* |
| **Appendices** |  |
| Appendix S1. Survey tool | *pag. 18* |
| Appendix S2. Pub-Med Citable Collaborators | *pag. 24* |

**Supplementary Methods**

**Figure S1**. The embedded mixed-method design.


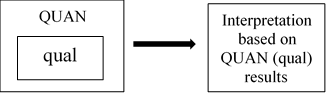


QUAN, Quantitative data set; qual, qualitative data set

Note: Uppercase denotes the primary method (QUAN), while lowercase indicates the secondary/supportive method (qual).

| **Figure S2**. Geographical distribution of survey respondents by country.  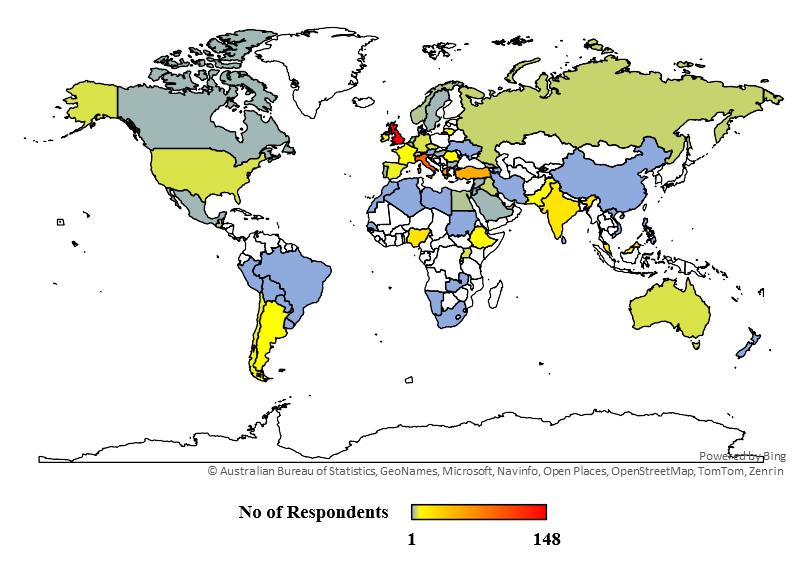 |
| --- |
| 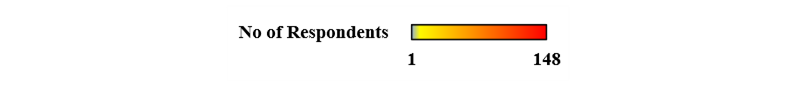 |

| **Figure S3**. Geographical distribution of the number of well-leg compartment syndrome cases reported by country.  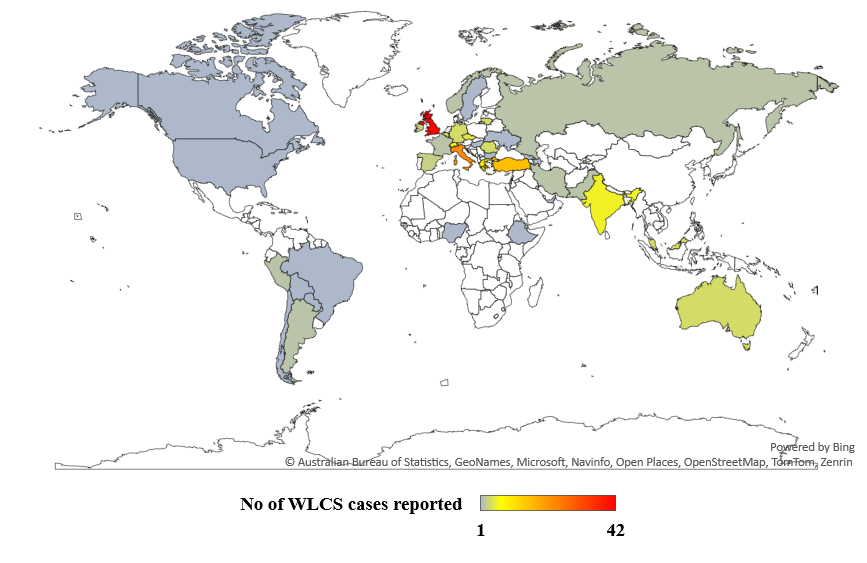 |
| --- |
| 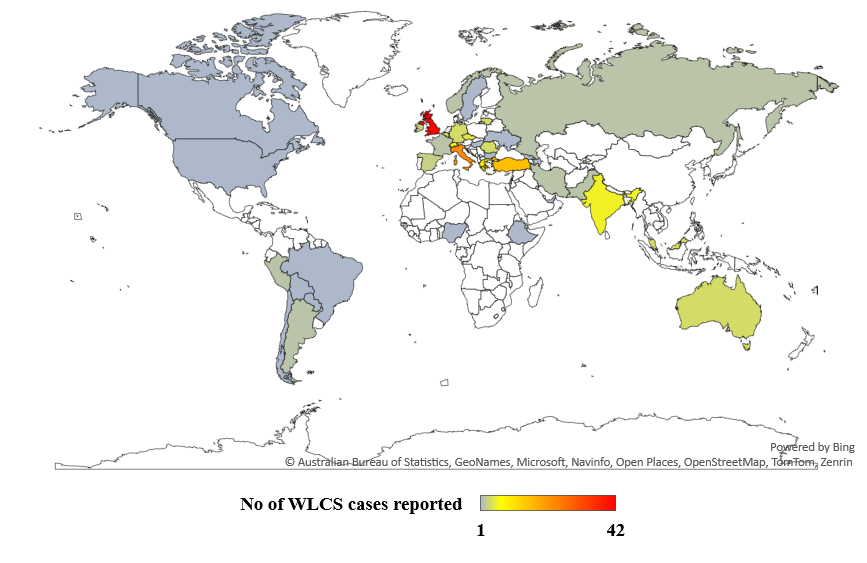 |

**Figure S4**. Distribution of the reported occurrence of well-leg compartment syndrome across ‘Duration of uninterrupted HDTL’ and ‘Length of MIS Experience’.

**A.** Bar chart showing the distribution of reported WLCS occurrence across different levels of ‘Duration of uninterrupted HDTL’. ‘4<hours’ responses included *1 hour*, *90 minutes*, and variable durations informed by stage of procedure. **B**. Bar chart showing the distribution of reported WLCS occurrence across different levels of ‘Length of MIS experience’.

WLCS, Well-leg compartment syndrome; HDTL, Head-down tilt lithotomy; MIS, Minimally invasive surgery.

**Supplementary Tables**

**Table S1**. Checklist for Reporting Of Survey Studies (CROSS) Checklist.

| **Section/topic** | **Item** | **Item description** | **Reported on page #** |
| --- | --- | --- | --- |
| **Title and abstract** | | |  |
| Title and abstract | 1a | State the word “survey” along with a commonly used term in title or abstract to introduce the study’s design. | 1 |
|  | 1b | Provide an informative summary in the abstract, covering background, objectives, methods, findings/results, interpretation/discussion, and conclusions. | 2-3 |
| **Introduction** | | |  |
| Background | 2 | Provide a background about the rationale of study, what has been previously done, and why this survey is needed. | 4 |
| Purpose/aim | 3 | Identify specific purposes, aims, goals, or objectives of the study. | 4 |
| **Methods** | | |  |
| Study design | 4 | Specify the study design in the methods section with a commonly used term (e.g., cross-sectional or longitudinal). | 5 |
|  | 5a | Describe the questionnaire (e.g., number of sections, number of questions, number and names of instruments used). | 6 |
| Data collection methods | 5b | Describe all questionnaire instruments that were used in the survey to measure particular concepts. Report target population, reported validity and reliability information, scoring/classification procedure, and reference links (if any). | 5-6 |
|  | 5c | Provide information on pretesting of the questionnaire, if performed (in the article or in an online supplement). Report the method of pretesting, number of times questionnaire was pre-tested, number and demographics of participants used for pretesting, and the level of similarity of demographics between pre-testing participants and sample population. | 6 |
|  | 5d | Questionnaire if possible, should be fully provided (in the article, or as appendices or as an online supplement). | Online supplement (S3) |
| Sample characteristics | 6a | Describe the study population (i.e., background, locations, eligibility criteria for participant inclusion in survey, exclusion criteria). | 5 |
|  | 6b | Describe the sampling techniques used (e.g., single stage or multistage sampling, simple random sampling, stratified sampling, cluster sampling, convenience sampling). Specify the locations of sample participants whenever clustered sampling was applied. | 5 |
|  | 6c | Provide information on sample size, along with details of sample size calculation. | n/a |
|  | 6d | Describe how representative the sample is of the study population (or target population if possible), particularly for population-based surveys. | n/a |
| Survey administration | 7a | Provide information on modes of questionnaire administration, including the type and number of contacts, the location where the survey was conducted (e.g.,outpatient room or by use of online tools, such as SurveyMonkey). | 6 |
|  | 7b | Provide information of survey’s time frame, such as periods of recruitment,  exposure, and follow-up days. | 6 |
|  | 7c | Provide information on the entry process:  –>For non-web-based surveys, provide approaches to minimize human error in data entry.  –>For web-based surveys, provide approaches to prevent “multiple participation” of participants. | n/a |
| Study preparation | 8 | Describe any preparation process before conducting the survey (e.g., interviewers’ training process, advertising the survey). | n/a |
| Ethical considerations | 9a | Provide information on ethical approval for the survey if obtained, including informed consent, institutional review board [IRB] approval, Helsinki declaration, and good clinical practice [GCP] declaration (as appropriate). | 5 |
|  | 9b | Provide information about survey anonymity and confidentiality and describe what mechanisms were used to protect unauthorized access. | 6 |
| Statistical analysis | 10a | Describe statistical methods and analytical approach. Report the statistical software that was used for data analysis. | 6-7 |
|  | 10b | Report any modification of variables used in the analysis, along with reference (if available). | n/a |
|  | 10c | Report details about how missing data was handled. Include rate of missing items, missing data mechanism (i.e., missing completely at random [MCAR], missing at random [MAR] or missing not at random [MNAR]) and methods used to deal with missing data (e.g., multiple imputation). | n/a |
|  | 10d | State how non-response error was addressed. | n/a |
|  | 10e | For longitudinal surveys, state how loss to follow-up was addressed. | n/a |
|  | 10f | Indicate whether any methods such as weighting of items or propensity scores have been used to adjust for non-representativeness of the sample. | n/a |
|  | 10g | Describe any sensitivity analysis conducted. | n/a |
| **Results** | | |  |
| Respondent characteristics | 11a | Report numbers of individuals at each stage of the study. Consider using a flow diagram, if possible. | Online supplement  (Table S4) |
|  | 11b | Provide reasons for non-participation at each stage, if possible. | Online supplement  (Table S4) |
|  | 11c | Report response rate, present the definition of response rate or the formula used to calculate response rate. | Online supplement  (Table S4) |
|  | 11d | Provide information to define how unique visitors are determined. Report number of unique visitors along with relevant proportions (e.g., view proportion, participation proportion, completion proportion). | Online supplement  (Table S4) |
| Descriptive results | 12 | Provide characteristics of study participants, as well as information on potential confounders and assessed outcomes. | 7;  Table 1 |
| Main findings | 13a | Give unadjusted estimates and, if applicable, confounder-adjusted estimates along with 95% confidence intervals and p-values. | 7 – 9 |
|  | 13b | For multivariable analysis, provide information on the model building process, model fit statistics, and model assumptions (as appropriate). | n/a |
|  | 13c | Provide details about any sensitivity analysis performed. If there are considerable amount of missing data, report sensitivity analyses comparing the results of complete cases with that of the imputed dataset (if possible). | n/a |
| **Discussion** | | |  |
| Limitations | 14 | Discuss the limitations of the study, considering sources of potential biases and imprecisions, such as non-representativeness of sample, study design, important uncontrolled confounders. | 13 – 14 |
| Interpretations | 15 | Give a cautious overall interpretation of results, based on potential biases and  imprecisions and suggest areas for future research. | 10 – 14 |
| Generalizability | 16 | Discuss the external validity of the results. | 14 |
| **Other sections** | | |  |
| Role of funding source | 17 | State whether any funding organization has had any roles in the survey’s design, implementation, and analysis. | 1 |
| Conflict of interest | 18 | Declare any potential conflict of interest. | 1 |
| Acknowledgements | 19 | Provide names of organizations/persons that are acknowledged along with their contribution to the research. | n/a |

**Table S2**. Respondent progress

| p.1 | p.2 | p.3 | p.4 | p.5 | p.6 | p.7 | p.8 | p.9 | p.10 | p.11 | **Total** |
| --- | --- | --- | --- | --- | --- | --- | --- | --- | --- | --- | --- |
| 1768 | 84 | 35 | 1 | 2 | 0 | 37 | 4 | 12 | 6 | 595 | **2509** |

*p - survey page*

This respondent progress table shows the last page visited by respondents before they dropped out of the survey. Owing to the convenience sampling method employed in this survey, unique visits were not tracked. Consequently, the progress table above displays how many times each survey page was viewed. It is worthy of note then that the same individual may have accessed the same pages more than once.

In total, 2509 visits were recorded, of these, 595 individuals (~23.7%) submitted their responses, which were subsequently screened and analysed.

**Table S3.** Characteristics of countries.

|  | **Respondents** | | **Ever encountered WLCS** | | **Number of reported WLCS cases** | | **Economy*** |
| --- | --- | --- | --- | --- | --- | --- | --- |
|  | ***n*** | **%** | ***n*** | **%** | ***n*** | **%** |  |
|  | **595** | **100.0** | **122** | **100.0** | **170** | **100.0** |  |
| United Kingdom | 148 | 24.9 | 29 | 23.8 | 42 | 24.7 | HIC |
| Italy | 95 | 16.0 | 15 | 12.3 | 23 | 13.5 | HIC |
| Greece | 68 | 11.4 | 8 | 6.6 | 10 | 5.9 | HIC |
| Turkey | 52 | 8.7 | 15 | 12.3 | 16 | 9.4 | MIC |
| India | 22 | 3.7 | 3 | 2.5 | 6 | 3.5 | MIC |
| Nigeria | 22 | 3.7 | 1 | 0.8 | 1 | 0.6 | MIC |
| Malaysia | 17 | 2.9 | 2 | 1.6 | 4 | 2.4 | MIC |
| Ethiopia | 8 | 1.3 | 1 | 0.8 | 1 | 0.6 | LIC |
| Lithuania | 7 | 1.2 | 3 | 2.5 | 4 | 2.4 | HIC |
| Romania | 7 | 1.2 | 3 | 2.5 | 4 | 2.4 | HIC |
| France | 7 | 1.2 | 2 | 1.6 | 2 | 1.2 | HIC |
| Argentina | 7 | 1.2 | 1 | 0.8 | 2 | 1.2 | MIC |
| Pakistan | 7 | 1.2 | 1 | 0.8 | 2 | 1.2 | MIC |
| Ireland | 6 | 1.0 | 2 | 1.6 | 3 | 1.8 | HIC |
| Spain | 6 | 1.0 | 2 | 1.6 | 3 | 1.8 | HIC |
| Chile | 6 | 1.0 | 1 | 0.8 | 1 | 0.6 | HIC |
| Czech Republic | 5 | 0.8 | 4 | 3.3 | 5 | 2.9 | HIC |
| Germany | 5 | 0.8 | 4 | 3.3 | 4 | 2.4 | HIC |
| Australia | 5 | 0.8 | 3 | 2.5 | 4 | 2.4 | HIC |
| United States | 5 | 0.8 | 1 | 0.8 | 1 | 0.6 | HIC |
| Switzerland | 4 | 0.7 | 2 | 0.8 | 7 | 4.1 | HIC |
| Belgium | 4 | 0.7 | 2 | 1.6 | 4 | 2.4 | HIC |
| Hong Kong SAR | 2 | 0.3 | 2 | 1.6 | 2 | 1.2 | HIC |
| Russian Federation | 4 | 0.7 | 1 | 0.8 | 2 | 1.2 | MIC |
| Norway | 3 | 0.5 | 1 | 0.8 | 2 | 1.2 | HIC |
| Iran | 1 | 0.2 | 1 | 0.8 | 2 | 1.2 | MIC |
| Peru | 1 | 0.2 | 1 | 0.8 | 2 | 1.2 | MIC |
| United Arab Emirates | 3 | 0.5 | 1 | 0.8 | 1 | 0.6 | HIC |
| Bulgaria | 2 | 0.3 | 1 | 0.8 | 1 | 0.6 | MIC |
| Canada | 2 | 0.3 | 1 | 0.8 | 1 | 0.6 | HIC |
| Georgia | 2 | 0.3 | 1 | 0.8 | 1 | 0.6 | MIC |
| Hungary | 2 | 0.3 | 1 | 0.8 | 1 | 0.6 | HIC |
| Sweden | 2 | 0.3 | 1 | 0.8 | 1 | 0.6 | HIC |
| Azerbaijan | 1 | 0.2 | 1 | 0.8 | 1 | 0.6 | MIC |
| Bolivia | 1 | 0.2 | 1 | 0.8 | 1 | 0.6 | MIC |
| Brazil | 1 | 0.2 | 1 | 0.8 | 1 | 0.6 | MIC |
| Paraguay | 1 | 0.2 | 1 | 0.8 | 1 | 0.6 | MIC |
| Ukraine | 1 | 0.2 | 1 | 0.8 | 1 | 0.6 | MIC |
| Uganda | 5 | 0.8 | / | / | / | / | LIC |
| Iraq | 4 | 0.7 | / | / | / | / | MIC |
| Netherlands | 4 | 0.7 | / | / | / | / | HIC |
| Egypt | 3 | 0.5 | / | / | / | / | MIC |
| Guatemala | 3 | 0.5 | / | / | / | / | MIC |
| Portugal | 3 | 0.5 | / | / | / | / | HIC |
| Lebanon | 2 | 0.3 | / | / | / | / | MIC |
| Mexico | 2 | 0.3 | / | / | / | / | MIC |
| Saudi Arabia | 2 | 0.3 | / | / | / | / | HIC |
| Syria | 2 | 0.3 | / | / | / | / | LIC |
| Algeria | 1 | 0.2 | / | / | / | / | MIC |
| Anguilla | 1 | 0.2 | / | / | / | / | HIC |
| Austria | 1 | 0.2 | / | / | / | / | HIC |
| China | 1 | 0.2 | / | / | / | / | MIC |
| Cyprus | 1 | 0.2 | / | / | / | / | HIC |
| Denmark | 1 | 0.2 | / | / | / | / | HIC |
| Jordan | 1 | 0.2 | / | / | / | / | MIC |
| Libya | 1 | 0.2 | / | / | / | / | LIC |
| Morocco | 1 | 0.2 | / | / | / | / | MIC |
| Namibia | 1 | 0.2 | / | / | / | / | MIC |
| New Zealand | 1 | 0.2 | / | / | / | / | HIC |
| Panama | 1 | 0.2 | / | / | / | / | HIC |
| Philippines | 1 | 0.2 | / | / | / | / | MIC |
| Qatar | 1 | 0.2 | / | / | / | / | HIC |
| Senegal | 1 | 0.2 | / | / | / | / | MIC |
| Singapore | 1 | 0.2 | / | / | / | / | HIC |
| South Africa | 1 | 0.2 | / | / | / | / | MIC |
| Sri Lanka | 1 | 0.2 | / | / | / | / | MIC |
| Sudan | 1 | 0.2 | / | / | / | / | LIC |
| Uruguay | 1 | 0.2 | / | / | / | / | HIC |
| Vietnam | 1 | 0.2 | / | / | / | / | MIC |
| West Bank | 1 | 0.2 | / | / | / | / | MIC |
| Zambia | 1 | 0.2 | / | / | / | / | MIC |

WLCS, Well-leg compartment syndrome; LIC, Low-income country; MIC, Middle-income country; HIC, High-income country

*Source: the [World Bank](https://datahelpdesk.worldbank.org/knowledgebase/articles/906519-world-bank-country-and-lending-groups), 2022 gross national income per capita, calculated using the World Bank Atlas method.

**Table S4.** Respondent-reported specific leg-rest protocols.

| We increase the leg-rest time in obese patients |
| --- |
| Flatten table every 3 hours (often with concurrent deflation of pneumoperitoneum) for at least 10mins |
| Reverse HDTL, lower down the leg |
| Reduce head down position, keep Flowtrons* on |
| Every 2 hours, if safe to take a break, patient is repositioned to a neutral position for 10 mins. Also, Flowtron* boots switched off in extreme head-down tilt. |
| No specific protocol, guided by surgeon preference. More prominent in centres where compartment syndrome has occurred |
| During left colonic and rectal resection, the position is changed during splenic flexure mobilisation to anti trend. Usually in the middle of the procedure for above 1 hour. |
| Never steep head down for longer than 2 hours at a time- flattened off |
| NHS trust protocol which is lead by the anaesthetist. The will notify surgeon at the appropriate time. Surgeon will then choose a convenient point in the operation as soon as possible after notification to remove head down tilt for 15 minutes. This is a trust wide policy |
| After 2 hours, leg checks performed every 30 mins until 4 hours when repositioning to patient neutral for 30 mins and then recommence lithotomy. |
| We just massage the legs |
| "Leg-rest"/repositioning after every 2.5-3 hours. 15-20 min each time |
| Check at 2.5h, if not going to finish in next 30mins to reposition |
| Although we use the head-down position, the legs of the patients are always in the leg rest position and are tilted only when the circular stapler is inserted in the anus |
| We just remove the Trendelenburg position for 10 minutes. |
| We switch from 25 degrees head-down tilt to neutral (0 degrees) supine position for 30 minutes then continue with HDTL for rest of the procedure. |

HDTL, Head-down tilt lithotomy, TEDS, Thromboembolic deterrent stockings

**Flowtron – an Intermittent pneumatic compression (IPC) device*

**Table S5.** Surgical procedures that preceded the reported cases of well-leg compartment syndrome cases.

| **Speciality** | **Procedure** | ***n*** |
| --- | --- | --- |
| Colorectal & General Surgery | Low anterior resection | 29 |
|  | Anterior resection | 11 |
|  | Total mesorectal excision | 10 |
|  | Laparoscopic low anterior resection | 8 |
|  | CRS + HIPEC | 5 |
|  | Robotic anterior resection | 5 |
|  | Abdominoperineal resection | 4 |
|  | Rectal cancer surgery | 4 |
|  | Laparoscopic anterior resection | 3 |
|  | Complex pelvic surgery (locally advanced rectal cancers) | 2 |
|  | Laparoscopic total colectomy | 2 |
|  | Laparoscopic ultra-low anterior resection | 2 |
|  | Open low anterior resection | 2 |
|  | Pelvic exenteration (open) | 2 |
|  | Bilateral groin dissection | 1 |
|  | Colon resection | 1 |
|  | Colproctectomy | 1 |
|  | Emergency colectomy for diverticular disease | 1 |
|  | Excision of rectum and formation of ileoanal pouch | 1 |
|  | Extensive lymphadenectomy and internal iliac vein bleed | 1 |
|  | Hartman reversal | 1 |
|  | High anterior resection for endometriosis | 1 |
|  | Laparoscopic abdominoperineal resection | 1 |
|  | Laparoscopic colectomy | 1 |
|  | Laparoscopic colon resection | 1 |
|  | Laparoscopic pan proctocolectomy | 1 |
|  | Laparoscopic rectal resection with colo-anal anastomosis | 1 |
|  | Laparoscopic right hemicolectomy | 1 |
|  | Laparoscopic total mesorectal excision | 1 |
|  | Left colectomy | 1 |
|  | Long proctocolectomy | 1 |
|  | Low anterior resection with colo-ano anastomosis | 1 |
|  | Low anterior resection with defunctioning loop ileostomy | 1 |
|  | Low rectal cancer surgery | 1 |
|  | MIS rectal resection | 1 |
|  | Open exenteration with bilateral pelvic and paraaortic node dissection | 1 |
|  | Pan-proctocolectomy | 1 |
|  | Pelvic exenteration | 1 |
|  | Proctectomy and pouch | 1 |
|  | Rectal resection | 1 |
|  | Recurrent pelvic sarcoma | 1 |
|  | Restorative proctocolectomy | 1 |
|  | Restorative proctocolectomy with ileal-pouch anal anastomosis for FAP | 1 |
|  | Reversal of Hartmann’s with an extensive prolonged pelvic dissection | 1 |
|  | Robotic abdominoperineal resection | 1 |
|  | Robotic intersphincteric resection | 1 |
|  | Robotic low anterior resection | 1 |
|  | Robotic low anterior resection-hand sewn coloanal anastomosis | 1 |
|  | Sigmoid cancer surgery | 1 |
|  | Sigmoid resection | 1 |
|  | Total colectomy | 1 |
|  | Total exenteration with high cortical sacrectomy | 1 |
|  | Total pelvic exenteration | 1 |
|  | Tumour excision | 1 |
|  | Ultra-low anterior resection | 1 |
| Urology | Robotic radical prostatectomy | 3 |
|  | Laparoscopic prostatectomy (in early learning curve) | 1 |
|  | Robot-Assisted Laparoscopic Prostatectomy | 1 |
| Obstetrics and Gynaecology | Laparoscopic hysterectomy for a very large uterus (> 2,5kg) | 1 |
|  | Complex interdisciplinary endometriosis surgery | 1 |
|  | Complex hysterectomy for large fibroid in previous 3 LSCS. | 1 |
|  | Açık histerektomi (Open hysterectomy) | 1 |
|  | Colectomy, hysterectomy | 1 |
|  | Gynaecological endometriosis resection laparoscopic with sigmoid segment resection (*reported by colorectal/general surgeon*) | 1 |

CRS – Cytoreductive surgery, HIPEC – Hyperthermic Intraperitoneal Chemotherapy, LSCS – Lower Segment Caesarean Section, MIS – Minimally invasive surgery, FAP – Familial Adenomatous Polyposis

**Table S6**. Representative quotes from free-text survey responses. Categories were generated from the qualitative data provided by respondents, representing their practices and perspectives regarding HDTL and WLCS.

| **Subcategory** | **Representative quotes** |
| --- | --- |
| **Category 1: ‘Leg-rest’/patient repositioning as a preventive measure** | |
| No formal (*institutional)* policy | “No formal protocol”  “Legs are straightened whenever feasible”  “No [specific protocol], it’s more ad hoc at convenient times of the case”  “No specific protocol, guided by surgeon preference” |
| Variability in practice | “Leg-rest"/repositioning after every 2.5-3 hours. 15-20 min each time”  “2h legs up means 30min legs down”  “5 minutes leg position after 3 hours of surgery”  “Leg rest mandatory for any procedure lasting longer than 4 hours for 5 minutes”  “Every 2 hours, take tilt off for 15 minutes” |
| Stage-informed protocol | “During left colonic and rectal resection, the position is changed during splenic flexure mobilisation to anti-trend. Usually in the middle of the procedure for above 1 hour”  “Variable depending on progress of case and feasibility of repositioning…” |
| Multiple measures  (Leg-rest + other) | “After 2 hours, leg checks were performed every 30 mins until 4 hours when repositioning to patient neutral for 30 mins and then recommence lithotomy”  “Flatten table every 3 hours (often with concurrent deflation of pneumoperitoneum) for at least 10mins”  “…leg checks every 30mins after 2hrs, mandatory leg rest at 4hrs”  “Putting patient head up and leg check every 4 hours” |
| Team approach | “NHS trust protocol which is led by the anaesthetist. They will notify the surgeon at the appropriate time. Surgeon will then choose a convenient point in the operation as soon as possible after notification to remove head-down tilt for 15 minutes. This is a trust-wide policy”  “We encouraged senior members of the team to help with patient positioning as a protocol…”  “Empower the team to remind you [about leg-rest] as a surgeon”  “Theatre staff education [required]” |
| **Category 2:** **WLCS experience impacts perception and practice** | |
| Rare complication | “In 20 y activity, I only heard about one case of compartment syndrome related to positioning”  “In my personal experience of 21 years, I have not had a single case of WLCS even though MIS with HDTL is routine practice at my working place”  “Very rare, seen one in an open case … in more than 2000 MIS pelvic procedures”  “We haven’t [seen] cases of Well-leg compartment syndrome in our more than 580 MIS Colorectal surgeries” |
| Attitude and perception | “Watch out [for the] legs!”  “Never steep head down for longer than 2 hours at a time- flattened off”  “Put a protocol in place”  “Must be on the consent form”  “Seems to be an overstated risk - never seen it…”  “There is no awareness of this condition and even if it develops, it goes unnoticed”  “…WLCS is uncommon for individual surgeons to encounter hence the complacency among surgeons to find measures to reduce it”  “In my practice of 30 yrs of MIS surgery … No well leg syndrome. Please do not waste time in a rare condition” |
| Practice | “[Leg-rest protocols are] More prominent in centres where compartment syndrome has occurred (I have only heard of one case in my career so far)”  “Have worked somewhere they used flat split legs for most of the colorectal and only put legs up at the end *as they had a compartment syndrome in the past*. Added to operative time though”  “In [hospital name] we have never seen one. And to be fair *never done leg repositioning for this* but whole body reposition just for intraoperative surgical field clarity” |
| **Category 3:** **Need for further research** | |
| Awareness | “Need to raise awareness and this becoming part of the peri-operative check”  “Surgical community needs to be fully aware of the well-leg compartment syndrome and should improve our pre-emptive measures”  “Awareness of WLCS for all surgeons performing surgery in HDTL position is essential”  “This is novel and ground-breaking as I have not heard the term before” |
| Need for clearly communicated standardised protocol | “I recently read about WLCS. I am not sure whether I have missed cases in the past. There is no clear criteria of when to refer to orthopaedics - it may be difficult to convince the orthopaedics registrar to come and see the patient if I am not able to convey my thoughts clearly. I feel that most of my colleagues, including me, will not be able to recognise WLCS”  “Best practice should become widely communicated”  “Evidence-based guidance from ACPGBI (or like) and encourage hospitals to have a SOP for positioning major MIS colorectal surgery” |
| Enthusiasm to contribute to further research | “We are a high-volume unit and will be happy to pilot interventions and input into SOP/guidelines”  “Preventing this serious complication can be approached systematically using retrospective data, audit data, and the latest research evidence to create a standardized protocol”  “I would love to contribute data if there is such a multicentre collaborative study”  “This is an area of great interest since the efficiency of minimally invasive surgery is improved by more head-down tilt but anaesthetic concern about the risk of complications is impacted by the lack of evidence base in this area. There is quite a lot of easy research which could answer these questions” |
| Suggestions for future research | “Compare the practice in the developing countries with the one in developed countries”  “Would find it very useful to know the actual incidence of this”  “Good to know the incidence (rarity) of WLCS in colorectal practice” |
| **Category 4: Considerations for practice** | |
| Consent | “Pelvic surgeons must give more information to the patients about WLCS”  “[WLCS] Must be on the consent form”  “To inform the patient and relatives before the operation about WLCS”  “Well-leg compartment syndrome should be considered in the counselling of our women before surgery”  “We now consent patient explicitly for neurovascular injury incl. severe long-lasting damage for all digestive surgery, quoting an incidence of <1%” |
| Pre and post-operative preventive measures | “2-week bike exercise - morning 20 min, afternoon 40 min; Breathing exercise 30-30-30” – *prehabilitation*  “…check legs routinely at the end of the procedure and in recovery”  “Routine clinical examination of the patients' legs post operatively”  “Department made aware of leg rest and checking post-op for any leg changes in recovery”  “Enhanced recovery after surgery (ERAS) protocol including early mobilization” |
| Alternative surgical positions | “We have introduced Extraperitoneal colorectal surgery … to exactly address the problem of head down position. Patients remain in a supine position which reduces their airway pressure and potentially reduces WLCS…We need to think extraperitoneally”  “Using the French position for non-transanal surgery (e.g. stapled anastomosis) offers the same accessibility, lowering the risk for WLCS”  “Now we changed to supine head down split leg position for all robotic radical prostatectomy cases. Also for robotic radical cystectomy, if urethrectomy is not needed in the same session” |

HDTL, Head-down tilt lithotomy; WLCS, Well-leg compartment syndrome; SOP, Standard operating procedure; ACPGBI, Association of coloproctology of Great Britain and Ireland.

**Appendix S1. Survey tool**

**Survey map**

| 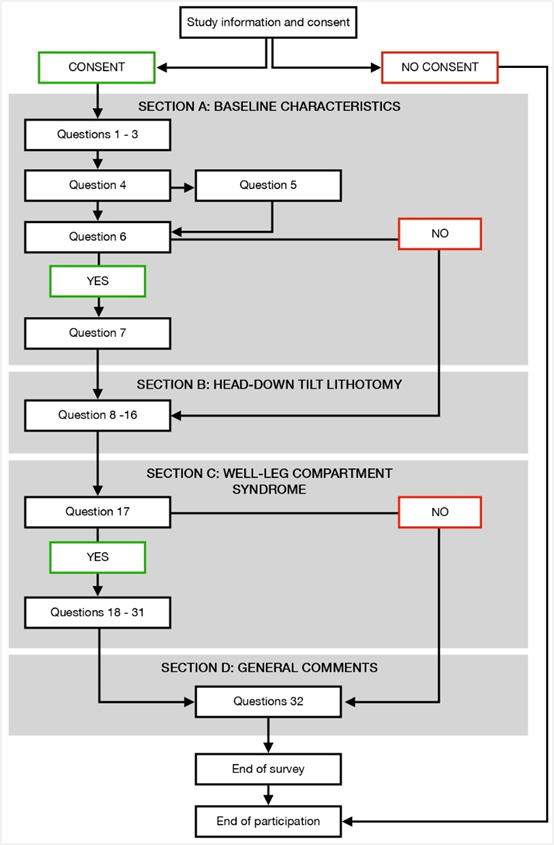 |
| --- |

**Participant Information Sheet v.3 (04/07/2023)**

**Study title**: Head-down tilt lithotomy position and well-leg compartment syndrome: an international survey of current practice trends

**Rationale and Aim:**

Minimally invasive surgery (MIS) has rapidly become the gold standard for abdominopelvic surgery. MIS requires specific criteria, such as unique patient positioning, to be efficient and safe. Head-down tilt lithotomy (HDTL) is commonly employed during colorectal, gynaecological, and urological MIS. This position allows for sufficient surgical access to the area of interest as abdominal organs are displaced away from the site by gravity. Prolonged HDTL is associated with unique and potentially severe adverse events including well-leg compartment syndrome (WLCS), ischaemic optic neuropathy and peripheral neuropathies. Incidence of these adverse events is expected to increase with the current rapid adoption of MIS, pushing the need for the surgical community to better understand and address them. To date, there is no shared consensus on optimal perioperative preventive strategies when HDTL is utilised.

This survey aims to assess current global real-world HDTL practice trends and evaluate the prevalence of and management strategies for WLCS. This study is the first phase of a broader project to inform expert-driven evidence-based guidance to improve patient safety when HDTL is employed during abdominopelvic MIS.

**Participation and Eligibility:**

We would like to invite you to take part in this study by completing a short survey about your current practices and clinical experience concerning HDTL position and WLCS. To participate in this study, you must meet the following criteria:

1. Be a consultant/attending, clinical fellow **or** career-grade specialist trainee (registrar/resident).
2. Routinely performing surgeries with the use of the head-down tilt lithotomy position **(please see definition below)**.
3. Specialities of interest: colorectal surgery, general surgery, lower & upper GI surgery, obstetrics & gynaecology, and urology.

Please answer the survey questions to the best of your knowledge. The survey should only take approximately **5 minutes** to complete. As partial compensation for participation, we would like to offer all respondents the opportunity to be listed as **collaborators (PubMed citable)** in the resulting publication. If you wish to join the Study Group, please follow the link provided on the last page of this survey.

Participation in this study is entirely voluntary and the responses provided will be anonymous. You can choose to withdraw from the study at any time before submission of the survey without giving a reason. However, please note that after submission, consent cannot be withdrawn.

No person-identifiable information will be required in this study. All data will be stored securely on a dedicated Google Drive, access permission to which will be restricted to only research team members as needed for the purposes of this study.

We will aim to publish study findings in peer-reviewed research journals and present them at local and international conferences. In line with current best practice to promote research transparency and to maximise the clinical/scientific benefits of this study, anonymous data will be shared publicly at the end of this project and made open access under a CC-BY licence. This means anyone (from the general public) may use this anonymised data for any purpose, providing that they credit the research team as the original creators.

**Definition of Terms**

**Head-down tilt lithotomy (HDTL) position** - patient maintained in lithotomy or Lloyd-Davies position with head-down tilt ≥15°. Also known as Lloyd-Davies position with Trendelenburg. 'Prolonged' is understood as HDTL maintained for >60 minutes.

**Well-leg compartment syndrome (WLCS)** - acute lower limb compartment syndrome that develops in an uninjured extremity usually in the absence of pre-existing vascular disease. Typically, this condition is preceded by prolonged surgery in the lithotomy position.

**“Leg-rest”** (patient repositioning) - lowering of the patient's legs below the level of the heart or the supine position.

* *while completing the survey, please click “more info” under each question to see definitions*

**Funding:**

This study forms part of a wider research project by the Faculty of Science and Health, University of Portsmouth and the Physiological Society in collaboration with Portsmouth Hospitals University NHS Trust examining the effects of prolonged head-down tilt lithotomy on lower limb haemodynamics.

**Ethical approval**:

This study has been reviewed by the Science and Health Faculty Ethics Committee, University of Portsmouth (Reference Number: SHFEC 2023-057).

**Contacts for further information**:

If you would like more information or have any query, concern or complaint about this study please contact the research team in the first instance using the details below:

- Dr Chukwuemeka Uzoma (Chief investigator, PhD researcher), University of Portsmouth - [chukwuemeka.uzoma@port.ac.uk](mailto:chukwuemeka.uzoma@port.ac.uk).
- Dr Maria Perissiou (Supervisor), University of Portsmouth - [maria.perissiou@port.ac.uk](mailto:maria.perissiou@port.ac.uk).

If concern or complaint remains unresolved by the researcher or their supervisor, please contact the head of school:

- Professor Richard Thelwell, School of Sport, Health and Exercise Science, University of Portsmouth - [richard.thelwell@port.ac.uk](mailto:richard.thelwell@port.ac.uk).

**Consent**

| I confirm that I have read and understood the information sheet dated 04/07/2023 (version 3) for the above study. | ◦ |
| --- | --- |
| I understand that my participation is voluntary and that I am free to withdraw at any time before submission of my responses without giving any reason. | ◦ |
| I understand that any information given by me may be used anonymously in future reports, articles or presentations by the research team. | ◦ |
| I understand that to maximise the benefits of this research, anonymous data (which does not identify me) will be publicly shared at the end of the project and made open access under a CC-BY licence. | ◦ |
| I confirm that I am a Consultant/Attending, Clinical Fellow or Career-grade specialist trainee (registrar/resident) routinely performing surgeries with the use of the head-down tilt lithotomy position. | ◦ |
| I agree to participate in this study. | ◦ |

**A. Baseline Characteristics_______**

1. **Age**
   - 20–29
   - 30–39
   - 40–49
   - 50–59
   - ≥ 60
   - I prefer not to say
2. **Gender ________________________**
3. **What is your surgical speciality?**

- Colorectal surgery
- General surgery
- Lower GI surgery
- Obstetrics and Gynaecology
- Upper GI surgery
- Urology
- Others (*specify*) _________________

1. **In which country do you practise? (Country drop-down list) _________________**
2. **Please state the closest city to your primary clinical workplace _________________**
3. **Do you perform minimally invasive surgery (MIS) in your practice?**
   - Yes
   - No
   - Others (*specify*) _________________

**MIS Experience**

1. **How long have you been performing MIS?**
   - <5 years
   - 5 - 10 years
   - >10 years

**B. Head-down Tilt Lithotomy**

1. **Do you utilise the head-down tilt lithotomy (HDTL) position in your practice?**
   - Yes
   - No
2. **Which of the following measures do you take to avoid neurovascular complications when using the HDTL position?** (choose all that apply)

- None
- Scheduled intraoperative “leg-checks”
- Intraoperative leg massage
- Intraoperative “leg-rest” (patient repositioning)
- Post-operative physical leg examination
- Other (specify) _________________

1. **After how many hours of uninterrupted HDTL is intraoperative “leg-rest” usually initiated in your practice?**

- Not applicable (Not routinely done)
- 2 hours
- 3 hours
- 4 hours
- Other (specify) _________________

1. **What is the average duration of an intraoperative “leg-rest” in your practice?**

- Not applicable (Not routinely done)
- 5 mins
- 10 mins
- 15 mins
- 20 mins
- >20 mins
- Other (specify) _________________

1. **If you use a specific "leg-rest"/patient repositioning protocol in your practice, please describe this protocol.**

**____________________________________________________________________**

1. **What type of leg support do you routinely use during surgeries in the HDTL position?**

(choose all that apply)

- Allen-stirrups (boot-like device supporting the calf)
- Posterior knee supports
- Ankle slings
- Other (specify) _________________

1. **Which method/s of venous thromboembolism (VTE) prophylaxis do you routinely use for these procedures?** (choose all that apply)

- Thromboembolic deterrent (TED) stockings
- Intermittent pneumatic compression devices (e.g., Flowtron®)
- Pharmacological prophylaxis
- Others (*specify*) _________________

1. **Do you regularly inform patients undergoing procedures in the HDTL position about the risk of well-leg compartment syndrome (WLCS)?**

- Yes
- No

1. **In your opinion, what risk factors are important for the development of WLCS?** (choose all that apply)

- Obesity
- Peripheral Arterial Disease
- Perioperative blood loss
- Diabetes
- Smoking
- Early learning curve case
- Operative time
- Other (*specify*) _________________

Do you have any additional comments about Head-down tilt lithotomy position in your practice?

________________________________________________________________________________

**C. Well-leg Compartment Syndrome**

1. **Have you ever encountered a case of well-leg compartment syndrome (WLCS) in your practice?**
   - No
   - Yes
2. **How many cases of WLCS have you encountered in your practice?**

- 1
- 2
- 3
- if >3 cases, please specify: _________________

1. **Was the case Unilateral or bilateral?**

- Unilateral
- Bilateral
- if >1 case, please specify: Case 2:_______; Case 3:_______

1. **After which procedure did this WLCS case(s) occur?** ______________________________________________________

if >1 case, please specify:

Case 2:___________________________________________________________________;

Case 3:___________________________________________________________________

1. **How often do you perform these surgical procedures per year?**

- < 20
- 20 - 50
- 50 - 100
- > 100

1. **On average, how often are these procedures performed annually in your hospital/institution within your speciality (i.e. by you and other colleagues)?**

- < 100
- 100 - 200
- 200 - 500
- > 500
- Other (specify) _________________

1. **What was the duration of the procedure?**

- < 2 hours
- 2 - 6 hours
- 6 - 10 hours
- > 10 hours
- if >1 case, please specify: Case 2:_______; Case 3:_______

1. **Which surgical approach was used for this procedure?**

- Robotic
- Laparoscopic
- Open/laparotomy
- Other (*specify*) _________________
- if >1 case, please specify: Case 2:_______; Case 3:_______

1. **Which intraoperative patient position was utilised for this procedure?**

- Lithotomy
- Lloyd-Davies
- Lithotomy + Trendelenburg (Head-down tilt lithotomy)
- Supine
- Other (specify) _________________

1. **To the best of your knowledge, when were the first symptoms of WLCS observed?**

- <2 hours post-op
- 2 - 6 hours post-op
- 6 - 24 hours post-op
- >24 hours post-op
- Others (specify) _________________

1. **How was the diagnosis established?** (choose all that apply)

- Clinical assessment
- Direct compartment pressure measurement
- Serum creatine kinase (CK)
- Other (specify) _________________
- if >1 case, please specify: Case 2:_______; Case 3:_______

1. **What was the time interval from diagnosis to intervention?**

- < 1 hour
- 1 - 5 hours
- 5 - 10 hours
- > 10 hours (specify) _________________
- if >1 case, please specify: Case 2:_______; Case 3:_______

1. **How was the case managed?**

- Conservative treatment (without fasciotomy)
- 1-3 compartment fasciotomy
- 4-compartment fasciotomy
- No treatment
- Other (specify) _________________
- if >1 case, please specify: Case 2:_______; Case 3:_______

1. **What was the final outcome?**

- Full recovery
- Paresis, pain and sensory deficit
- Significant muscle loss
- Foot drop
- Limb amputation
- Other (specify) _________________
- if >1 case, please specify: Case 2:_______; Case 3:_______

1. **Were there any important learning points from the follow-up of this/these cases?**

__________________________________________________________________________

**Do you have any additional comments about well-leg compartment syndrome?** _______

**D. General Comments**

**32. If you have any additional comments and/or information about the subject of this research - Head-down tilt lithotomy position and Well-leg compartment syndrome, please feel free to write them below:** _______________________________________________________________________

**E. Thank you**

**Thank you for taking the time to participate in this study.**

To join the Study Group as a collaborator please follow this link: <https://portsmouth.onlinesurveys.ac.uk/the-help-project-study-group>

The results of this survey will provide a snapshot of current clinical practice when head-down tilt lithotomy position is utilised during abdominopelvic MIS as well as vital information about the prevalence and management of well-leg compartment syndrome. These findings will play a crucial role in informing evidence-based expert recommendations on HDTL and WLCS.

The survey findings will be published in peer-reviewed research journals and presented at international conferences. Your data will always remain anonymous, no person-identifiable information will be included in the dataset.

If you have any queries about the study, please contact the research team - Dr Chukwuemeka Uzoma ([chukwuemeka.uzoma@port.ac.uk](mailto:chukwuemeka.uzoma@port.ac.uk)) or Dr Maria Perissiou ([maria.perissiou@port.ac.uk](mailto:maria.perissiou@port.ac.uk)).

If you feel you need any emotional or mental support due to any issues raised within the survey, please contact:

- Samaritans: You can call free on 116 123 (24/7 helpline), email [jo@samaritans.org](mailto:jo@samaritans.org), visit <https://www.samaritans.org/>
- British Red Cross National Support Line: 0808 196 3651 (Monday to Friday - 10am to 5pm, Wednesday - 10am to 8pm), <https://www.redcross.org.uk/get-help/support-line>
- For support outside of the UK, contact your nearest National Red Cross and Red Crescent Society at <https://www.ifrc.org/national-societies-directory>

**Survey PDF version:** [**here**](https://drive.google.com/file/d/1_nrYRht45Mj9uqH1MQ8isv__ZKYzBon6/view?usp=sharing)

**Appendix S2.** Pub-Med Citable Collaborators

*HDTL-WLCS Global Survey Collaborating Group*

Aakansha Giri Goswami [AIIMS Rishikesh]; Aamir Aziz [Hillingdon Hospital NHS Trust]; Abdolazeem Elnour [Hywel Dda UHB]; Abdourahmane Ndong [Gaston Berger University]; Abdul Fattah Bin Abdul Hamid [Hospital Banting]; Abdullah Khan [Mateen Medical Center, University Town, Peshawar]; Abdullahi Musa Kulfi [Abubakar Tafawa Balewa University Teaching Hospital Bauchi Nigeria]; Adam Mylonakis [First Department of Surgery, Laiko General Hospital, National and Kapodistrian University of Athens, Greece]; Ademola Adeyeye [Afe Babalola University]; Adriana Liceaga Fuentes; Agnė Miknevičiūtė [Lietuvos Sveikatos Mokslų Universitetas Kauno Klinikos]; Ala Hassouneh [University Hospital of North Midland]; Alaa El-Hussuna [OpenSourceResearch Organisation]; Alazar Berhe Aregawi [Hawassa University Comprehensive Specialized Hospital]; Alberto Aiolfi [IRCCS Ospedale Galeazzi-Sant’Ambrogio]; Alberto Arezzo [Department of Surgical Sciences University of Turin]; Alberto Ignacio Herrando [Champalimaud Foundation]; Alberto Vannelli [Ospedale Valduce]; Alessia Malagnino [General and Emergency Surgery, A. Manzoni Hospital Lecco]; Alexander Julianov [Trakia Hospital, Stara Zagora, Bulgaria]; Alexandros Kozadinos [Laiko Hospital NKUA, Greece]; Alexis Theodorou [Hygeia Hospital & Aretaieio University Hospital, Athens Greece]; Ali Cihat Yildirim [Kütahya Health Sciences University]; Ali Guner [Karadeniz Technical University, Faculty of Medicine]; Ali Malik [East Suffolk & North Essex NHS Trust - Ipswich Hospital]; Ali Yasen Mohamedahmed [Royal Wolverhampton NHS Trust]; Alisina Bulut [Marmara University, Department of General Surgery]; Amedeo Antonelli [Università degli Studi Roma Tor Vergata]; Amir Botros [Queen Alexandra Hospital Portsmouth]; Amro Ahmed Mureb [King Hussein Cancer Center]; Andee Dzulkarnaen Zakaria [Department of Surgery, School of Medical Sciences & Hospital USM, Universiti Sains Malaysia, Kubang Kerian, Kelantan, Malaysia]; Andrea Divizia [Policlinico Tor Vergata]; Andrea Martina Guida [Policlinico Tor Vergata]; Andrea Romanzi [Valduce Hospital, Department of General Surgery, Como, Italy]; Andrew A. Gumbs [American Hospital Tblisi]; Andrew Craig Lynch [Sydney Adventist Hospital, Sydney Australia]; Andrew Day [Surrey and Sussex Healthcare NHS Trust]; Angelo Alessandro Marra [Fatebenefratelli Isola Tiberina - Gemelli Isola, Rome, Italy]; Angelo Parello [Ospedale Fatebenefratelli Gemelli Isola]; Anna Paspala [Department of Surgery, Evgenideio Hospital]; Anthony Lin [Department of Surgery & Anaesthesia, University of Otago, Wellington]; Antonio Castaldi [CHU Nîmes]; Antonio Luberto [Department of Biomedical Sciences, Humanitas University, Milan, Italy]; Antonio Simone Laganà [Department of Health Promotion, Mother and Child Care, University of Palermo, Italy]; Antonios Koutras [Alexandra Maternity Hospital, National and Kapodistrian University of Athens]; Arcangelo Picciariello [University “Aldo Moro” of Bari]; Argyrios Ioannidis [Athens Medical Center]; Aristeidis Papadopoulos [General Hospital of Nikaia, Greece]; Arshad Rashid [Government Medical College Srinagar]; Asad Ali Kerawala [Cancer Foundation Hospital]; Athanasios Marinis [3rd Department of Surgery, Tzaneio General Hospital, Piraeus, Greece]; Athanasios Pantelis [Mohak Bariatric and Robotic Surgical Center, Indore, MP, India]; Athanasios Syllaios [Laiko General Hospital]; Austin Acheson [University of Nottingham]; Avanish Saklani [Tata Memorial Hospital, Mumbai]; Ayandele Babajide Oladayo [Federal Medical Centre, Keffi, Nasarawa State, Nigeria]; Badri Kobalava [Tbilisi State Medical University]; Beata Hemmelova MD [Masaryk University Brno, Saint Anne's Hospital, Dept of Surgery, Brno Pekarska]; Beatrice Drago [Università degli Studi di Genova]; Binyam Yohannes [St Paul's Hospital Millennium Medical College]; Boby Sebastian [West Suffolk Hospital]; Carlo Ratto [Catholic University Rome Italy]; Casoni Pattacini Gianmaria [Department of General Surgery, Emergencies and New Technologies Baggiovara Civil Hospital Modena Italy]; Cem Emir Guldogan [Qatar Turkish Hospital]; Cezar Ionut Ciubotaru [“Carol Davila” University of Medicine of Pharmacy]; Chigozie Innocent Onyeze [NHS Tayside]; Christian Agbo Agbo [Benue State University Teaching Hospital Makurdi Nigeria]; Christina Bali [University Hospital of Ioannina]; Christina Fleming [Department of Colorectal Surgery, University Hospital Limerick]; Christina Kontopoulou [Aretaieion Hospital]; Christos Chouliaras [Athens Medical Center, Athens, Greece]; Cigdem Benlice [Ankara University]; Cihad Tatar [Acibadem Taksim Hospital]; Cihangir Akyol [Ankara University School of Medicine Department of Surgery]; Claudio Coco [Università Cattolica del Sacro Cuore - Fondazione Policlinico Universitario A.Gemelli - IRCCS Roma]; Colin Peirce [University Hospital Limerick]; Constantinos Nastos [3rd Department of Surgery, Attikon University Hospital]; Cristian Gallardo [Hospital Clínico San Borja Arriarán]; Cristiano Huscher [COBELLIS Clinic Surgical Oncology Robotic and New Technology]; Danette Wright [Blacktown and Mt Druitt Hospital]; Daniel Wilby [Portsmouth Hospitals University NHS Trust]; Danielle Brogden [Imperial College London]; Daunia Verdi [Department of General Surgery, Mirano Hospital, Venice, Italy]; David Merlini [ASST Rhodense - Ospedale di Garbagnate]; David Watt [University Hospital Crosshouse]; Davide Pertile [I.R.C.C.S. Ospedale Policlinico San Martino - Genova]; Dermot Burke [University of Leeds]; Despotidis Markos [First Department of Surgery, National and Kapodistrian University of Athens, Laikon General Hospital, Athens, Greece]; Diego Sasia [Santa Croce and Carle Hospital, Cuneo]; Dimitrios Ntourakis [European University Cyprus]; Dimitrios Papaconstantinou [Third Department of Surgery]; Dimitrios Tsapralis [General Hospital of Ierapetra]; Dimitris P. Korkolis [Hellenic Anticancer Hospital “Saint Savvas”]; Dimosthenis Chrysikos [First Department of Propaedeutic Surgery, Hippocration Hospital, School of Medicine, University of Athens, Athens, Greece]; Diogo Carrola Gomes [Centro Hospitalar Universitário Lisboa Central]; Douglas Bowley [University Hospitals Birmingham NHS Foundation Trust]; Dragomir Dardanov [Hospital St. George Pernik]; Ebbe Billmann Thorgersen [OUH The Radium Hospital, Oslo, Norway]; Egemen Ozdemir [Istanbul University-Cerrahpaşa]; Ekhlas Jabber Kadhim [Baghdad Medical College]; Eleftherios Spartalis [REA Maternity Hospital]; Eleni Andriopoluou [Hellenic Red Cross Korgialeneio Benakeio NHS Greece]; Elisa Reitano [Department of Digestive and Endocrine Surgery, University of Strasbourg, France]; Elizabeth Li [University of Birmingham]; Ellen Van Eetvelde [UZ Brussel]; Emmanuel Akpo [Delta State University Teaching Hospital, Oghara]; Emre Gonullu [Sakarya University Faculty of Medicine, Department of Gastrointestinal Surgery]; Emre Gunay [Liv Hospital Vadi Istanbul]; Enver Tekin; Ewen Harrison [University of Edinburgh]; Eyup Murat Yilmaz [Aydin General Clinic]; Fabrizio Sammartano [Ssd Trauma Team, San Carlo Borromeo Trauma Center, ASST Santi Paolo e Carlo, Milan Italy]; Fang Yi Cheung [North Central and East London Deanery]; Farhanul Huda [AIIMS Rishikesh India]; Federica Di Marco [Ospedale Maggiore di Modica]; Feras AlJarad [Ninewells Hospital and University of Dundee]; Filippo Carannante [Colorectal Clinical and Research Unit, Fondazione Policlinico Campus Bio-Medico di Roma]; Firdaus Hayati [Department of Surgery, Faculty of Medicine and Health Sciences, Universiti Malaysia Sabah, Kota Kinabalu, Sabah, Malaysia]; Floris Boudewijn Poelmann [University Medical Center Groningen]; Fodor Decebal [“Regina Maria” Hospital Brasov, Romania]; Frances Dixon [Milton Keynes University Hospital]; Francesco Bianco [Ospedale San Leonardo AS-NA3sud, Castellammare di Stabia, Naples]; Francesco Pata [Department of Pharmacy, Health and Nutritional Sciences, University of Calabria, Rende, Italy]; Gabriela Arroyo Murillo [General Surgery Unit, Dolo Hospital, Ulss 3 Serenissima]; Gabriella Marchitelli [NHS Lothian]; Gabrielle H. van Ramshorst [Ghent University Hospital, Ghent, Belgium]; Gaia Colletti [University of Milan, Via Festa del Perdono 7, 20122 Milan, Italy]; Ganendra Paramasvaran [General Hospital Kuala Lumpur]; Georgios Fragulidis [Second Department of Propedeutic Surgery, School of Medicine, National and Kapodistrian University of Athens, Athens, Greece]; Georgios Rallis [ELPIS General Hospital of Athens, Greece]; Georgios Stravodimos [Evaggelismos Hospital, Athens, Greece]; Gerald David [Queen Alexandra Hospital, Portsmouth Hospitals University NHS Trust]; Giacomo Calini [Dept. of Medical and Surgical Sciences - DIMEC, Alma Mater Studiorum - University of Bologna, Bologna, Italy; Surgery of the Alimentary Tract, IRCCS Azienda Ospedaliero-Universitaria di Bologna, Bologna, Italy]; Giacomo Carganico [Università degli Studi di Genova]; Giampaolo Formisano [University of Milan, Dipartimento di Scienze della Salute]; Gianluca Cassese [Federico II University Hospital]; Gianluca Pellino [Colorectal Surgery, Vall d'Hebron University Hospital, Universitat Autonoma de Barcelona UAB, Barcelona, Spain]; Giorgio Bogani [Fondazione IRCCS Istituto Nazionale dei Tumori di Milano]; Giorgio Dalmonte [General Surgery Unit - University Hospital of Parma]; Giorgio Lisi [Department of Surgery, Sant'Eugenio Hospital, Rome, Italy]; Giovanni Cestaro [ASST VALLE OLONA - Ospedale di Gallarate]; Giulia Turri [University of Verona]; Giuseppe Brisinda [Fondazione Policlinico Universitario A Gemelli IRCCS Rome, Italy]; Giuseppe Cucinella [Department of Surgical, Oncological and Oral Sciences (Di.Chir.On.S.), University of Palermo, Palermo, Italy]; Giuseppe Frazzetta [Arnas Civico Palermo Chirurgia Oncologica]; Gonzalo P. Martin-Martin [Hospital Doctor López-Cano Cádiz]; Habeeb Olufemi Gbenga [University of Ilorin Teaching Hospital and University of Ilorin, Kwara State, Nigeria]; Haluk Kerim Karakullukcu [University of Health Science, Umraniye Education and Research Hospital]; Harald Krentel [Bethesda Hospital Duisburg]; Hasan Mukhtar [The Whittington Hospital]; Hashim E Elmansi Abdalla [General Surgery, New Cross Hospital, The Royal Wolverhampton NHS Trust]; Hemendra Kumar Mangal [LHDM Cancer Hospital]; Hossam Elfeki [Mansoura University Hospital]; Ian Daniels; Ibrahim Darwich [St. Marienkrankenhaus Siegen]; Ibrahim Umar Garzali [Aminu Kano Teaching Hospital]; Ifeanyichukwu Kelvin Egbuchulem [University College Hospital Ibadan, Nigeria]; Ilenia Merlini [San Benedetto del Tronto Hospital, AST Ascoli Piceno, Italy]; İlgar Ismayilov [Republican Treatment and Diagnostic Center, Azerbaijan]; İlknur Turan; Ioannis Katsaros [First Department of Surgery, National and Kapodistrian University of Athens, Laikon General Hospital]; Ioannis Virlos [Metropolitan General Hospital, Athens, Greece]; Ionut Negoi [Carol Davila University of Medicine and Pharmacy Bucharest, Emergency Hospital of Bucharest, Romania]; Irfan Ahmed [NHS Grampian]; Jacopo Andreuccetti [General Surgery 2, ASST Spedali Civili of Brescia]; James Glasbey [University of Birmingham]; James Olivier [Royal United Hospital Bath]; James Wheeler [Cambridge University Hospitals NHS Foundation Trust]; Jamil Ahmed [Northampton General Hospital]; Jan Cagaš [Department of Surgery, Hospital Třebíč, Czech Republic]; Jared Torkington [University Hospital of Wales, Cardiff]; Jayesh Sagar [Luton and Dunstable Hospital]; Jeremy Meyer [Division of Digestive Surgery, University Hospitals of Geneva, Geneva, Switzerland]; Jeremy Yuen-Chun Teoh [The Chinese University of Hong Kong]; Jin Jiun Mah [Queen Elizabeth Hospital]; John Afam-Osemene [Federal Medical Centre, Asaba, Nigeria]; Jonathan Lee [Department of Surgery, The Chinese University of Hong Kong]; Joris P. Bulte [Södra Alvborgs Sjukhus, Borås]; Joseph Mathew [HealthCare Global (HCG) Enterprises Ltd]; Justin Davies [Addenbrooke’s Hospital, Cambridge University NHS Foundation Trust, UK and University of Cambridge, Cambridge, UK]; Kapil Sahnan [Imperial College London]; Kashish Malhotra [Dayanand Medical College, India]; Kaushika Gunasekare [Department of Surgery, Faculty of Medicine, University of Peradeniya]; Kemal Erdinç Kamer [HEALTH SCIENCES UNIVERSITY / Izmir Tepecik Education And Research Hospital]; Khaled Rida [Alfardan Medical with Northwestern Medicine]; Kollaras C Vasileios [General Surgery Clinic, Thriasio Hospital, Magoula Attikis, Greece]; Konstantinos Apostolou [Athens Medical Center Palaio Faliro, Department of General and Endocrine Surgery]; Konstantinos Bouchagier [University Hospital of Patras]; Konstantinos Kopanakis [Thebes General Hospital, Greece]; Konstantinos Stratakis [1st Department of Surgery, General Hospital of Athens G. Gennimatas]; Kosachenko Mikhail [Hospital named V.V. Vinogradov, Moscow, Russia]; Kris Jourand [Cambridge University Hospital NHS Foundation Trust]; Krunal Khobragade [KIMS Kingsway Hospital, Nagpur]; Lasitha Bhagya Samarakoon; LAWAL Bashir Oladimeji [Lagoon Hospitals, Lagos, Nigeria]; Leandro Siragusa [University of Rome Tor Vergata]; Linardoutsos Dimitrios [Metropolitan General Hospital Athens]; Lopez-Lopez Victor [Clinic and University Virgen de la Arrixaca Hospital]; Luca Domenico Bonomo [General Surgery Unit, Rivoli Hospital, Italy]; Luca Pio [Pediatric Surgery Department, Groupement Hospitalier Paris Sud (GHUPS), Hôpital Bicêtre, Le Kremlin-Bicêtre, France]; Lucio Taglietti [ASST Valcamonica Esine, Italy]; Luigi Battaglia [Colorectal Surgery Unit, Fondazione IRCCS Istituto Nazionale dei Tumori, Milan, Italy]; Luigi Bonavina [University of Milan, Department of General and Foregut Surgery, IRCCS Policlinico San Donato]; Mah Muneer Khan [Khyber Medical College and Khyber Teaching Hospital, Peshawar, Pakistan]; Malcolm A West [Cancer Sciences, Faculty of Medicine, University of Southampton]; Marco Cannistra' [Ospedale San Giovanni di Dio - Crotone]; Marco Catarci [Surgical Unit, Sandro Pertini Hospital, Rome, Italy]; Marco Giacometti [Ospedale Civile di Guastalla (RE), AUSL-IRCCS di Reggio Emilia]; Maria Chiara Sighinolfi [ASST Santi Paolo e Carlo, Milan, Italy]; Maria Papadoliopoulou [Attikon University Hospital, Athens, Greece]; Maria Sotiropoulou [Evangelismos General Hospital]; Mario Trompetto [S Rita Clinic, Vercelli, Italy]; Marius Kryzauskas [Vilnius University, Faculty of Medicine]; Marius T. Paraoan [Wrightington, Wigan and Leigh Teaching Hospitals NHS Foundation Trust]; Mark A Potter [Western General Hospital, Edinburgh, NHS Lothian]; Mark R Brincat [Barts Health NHS Trust]; Marta Spalluto [Università degli Studi di Milano - Scuola di Specializzazione in Chirurgia Generale]; Martin Rutegård [Umeå University]; Mauro Podda [Department of Surgical Science, University of Cagliari, Italy]; Maximos Frountzas [First Propaedeutic Department of Surgery, Hippocration General Hospital of Athens]; Mehmet Ali Koç [Ankara University School of Medicine]; Mehmet Ömer Özduman [Gastrointestinal Surgery]; Mejudin Kedir Abdella [Worabe Comprehensive Specialised Hospital]; Mert Guler [Istanbul Research and Teaching Hospital]; Michael Spartalis [Sotiria General Hospital]; Michail Vailas [Laiko General Hospital]; Michel Adamina [Kantonsspital Winterthur, University of Basel]; Michele Ballabio [ASST Lodi - Ospedale Maggiore di Lodi]; Mohamed A Thaha [Queen Mary University of London]; Mohamed Arif Hameed Sultan [University Malaysia Sabah]; Mohamed Ebrahim [Gastrounit, Surgical Division, Copenhagen University Hospital Hvidovre, Denmark]; Mohammad Faraz Khan [Mater Misericordiae University Hospital, Dublin]; Mohammed Basheeruddin Inamdar [Manipal Hospital, Bangalore]; Mohammed Eid [Society of Surgical Oncology]; Mohana Raj Thanapal [Hospital Kuala Lumpur]; Mohd Syakir Mohd Azahar [Hospital Sultan Abdul Aziz Shah, UPM, Serdang, Selangor, Malaysia]; Morini Andrea [AUSL-IRCCS Reggio Emilia, Surgical Oncology Unit]; Mostafa Shalaby [Mansoura University]; Muhammad Nur Syamim bin Che Johan [Department of Surgery, Faculty of Medicine and Health Sciences, Universiti Malaysia Sabah, Kota Kinabalu, Sabah, Malaysia]; Muhammad Salman Shafique [Chesterfield Royal Hospital]; Muhammad Shamim [Dept of Surgery, College of Medicine, Prince Sattam bin Abdul Aziz University]; Muhammad Umar Younis [Mediclinic City Hospital Dubai]; Muhammer Ergenç [Marmara University School of Medicine]; Mukoro Duke George [Ahmadu Bello University Teaching Hospital, Zaria, Kaduna]; Murat Kalin; Mustafa Yener Uzunoglu [Department of General Surgery, Bursa City Hospital]; Naciye Cigdem Arslan [Istanbul Medipol University, Department of General Surgery]; Narimantas Evaldas Samalavicius [Department of Surgery, Klaipeda University Hospital, Klaipeda, Lithuania]; Nathan Curtis [Dorset County Hospital NHS Foundation Trust]; Navin Kumar [All India Institute of Medical Sciences, Rishikesh, India]; Nguyen Thanh Sang [Trung Vuong Hospital]; Nicholas Wong [East of England Deanery]; Nicola Cinardi [ARNAS Garibaldi, Catania]; Nicola de'Angelis [Unit of Colorectal and Digestive Surgery, Beaujon Hospital, Clichy]; Nicolas Flamey [UZ Leuven]; Nicolò Tamini [IRCCS San Gerardo, Monza]; Nikolaos Chatzizacharias [Queen Elizabeth Hospital Birmingham]; Nikolaos Koliakos [Digestive Surgery Department, CHU Saint-Pierre, Bruxelles, Belgium]; Nikolaos Machairas [2nd Department of Propaedeutic Surgery, NKUA, Athens, Greece]; Nikolaos V Michalopoulos [Attikon University Hospital, Athens, Greece]; Nnaemeka Eli [Newcastle upon Tyne]; Nurhilal Kızıltoprak [Istanbul Sultan Abdülhamid Han Research and Training Hospital]; Nuri Okkabaz [Atlas University School of Medicine]; Nurudeen Akinbami [University College Hospital, Ibadan]; Okechukwu Hyginus Ekwunife [Nnamdi Azikiwe University Teaching Hospital, Nnewi]; Omer Faruk Ozkan [University of Health and Science Abdulhamid Training and Research Hospital]; Omer Yalkin [Ozel Doruk Nilufer Hospital]; Omorodion Irowa [University of Benin Teaching Hospital]; Orçun Yalav [Çukurova University]; Orestis Ioannidis [4th Department of Surgery, Medical School, Aristotle University of Thessaloniki, General Hospital “George Papanikolaou,” Thessaloniki, Greece]; Pamela Milito [IRCCS Policlinico San Donato]; Panteleimon Vassiliu [Attikon University Hospital, NKUA]; Paolo Delrio [Colorectal Surgical Oncology, Istituto Nazionale dei Tumori di Napoli, Fondazione G Pascale IRCCS, Naples, Italy]; Pascal Herzog [Kantonsspital Baden]; Patricia Tejedor [University Hospital Gregorio Marañon, Madrid]; Paul Okeny [Department of Surgery, Makerere University College of Health Sciences]; Peter Ihnát [University Hospital Ostrava]; Peter Ikponmwosa Agbonrofo [University of Benin Teaching Hospital, Benin City]; Petr Vlček [1st Department of Surgery, St. Ann’s University Hospital, University of T.G. Masaryk, Brno, Czech Republic]; Philip H Pucher [Portsmouth Hospitals University NHS Trust]; Pietro Fransvea [Fondazione Policlinico Universitario A Gemelli IRCCS]; Prashant Naik [William Harvey Hospital]; Prem Thambi [James Cook University Hospital]; Promise Wichendu [Rivers State University Teaching Hospital, Port Harcourt]; Rachel McKinney [Wirral University Hospital Trust]; Rafael Garatea Grau [General Surgery and Surgical Oncology Coordinator]; Rafael Sanchez Salas [Department of Surgery, Division of Urology, McGill University, Canada]; Raffaele Galleano [Ospedale San Paolo, Savona, Italy]; Raimundo Izquierdo [Hospital DIPRECA]; Rajeev Peravali [Sandwell and West Birmingham Hospitals NHS Trust]; Rany Aoun [Centre Hospitalier de Béthune Beuvry, France]; Ravi Aggarwal [Imperial College London]; Rebecca Reid [Wirral University Teaching Hospital NHS Foundation Trust]; Reinaldo Isaacs [Caja de Seguro Social - Panamá]; Renan Carlo Colombari Monteiro [Hospital Gregorio Marañón]; Renato Gomes Campanati [Hospital das Clínicas, Federal University of Minas Gerais]; Rikesh Patel [Liverpool University Hospitals NHS Trust]; Roberto Sampietro [Ospedale Moriggia Pelascini, Gravedona, Italy]; Rogier Crolla [Amphia Breda, Netherlands]; Romulo R. Cabantac III [St. Luke's Medical Center, Philippines]; Ronald Mbiine [Department of Surgery, Makerere University College of Health Sciences]; Rubén Domínguez Azuaga; Saburi Oyewale [University of Ilorin Teaching Hospital, Ilorin]; Sajith Pankajavihar Sasi [The Christie NHS Foundation Trust]; Salih Müjdat Balkan [GENEL CERRAHİ]; Salomone Di Saverio [San Benedetto Del Tronto Madonna del Soccorso Hospital]; Samuel Stefan [Colorectal Surgery, NHS Portsmouth, UK]; Santoro Giulio Aniello [Third Referral Pelvic Floor Center, Regional Hospital Treviso, Italy]; See Boon Keong; Semra Demirli Atici [Acibadem Kent Hospital]; Sentilnathan Subramaniam [Hospital Queen Elizabeth, Sabah, Malaysia]; Seon Hahn Kim [Professor, Department of Surgery, Faculty of Medicine, Universiti Malaya, Kuala Lumpur, Malaysia]; Sergey Efetov [IM Sechenov First Moscow State Medical University]; Sergio M Navarro [University of Minnesota]; Sevcan Arzu Arinkan [Central Hospital Kristianstad]; Sezai Leventoglu [Gazi University School of Medicine]; Shafaque Shaikh [Aberdeen Royal Infirmary; University of Aberdeen]; Simon Middleton [Royal Berkshire Hospital]; Simone Manfredelli [General and Digestive Surgery, Hautepierre Hospital, Strasbourg]; Somprakas Basu [All India Institute of Medical Sciences, Rishikesh]; Spyridon Christodoulou [4th Department of Surgery, Attikon University Hospital, National and Kapodistrian University of Athens]; Spyridon Dritsas [Evaggelismos General Hospital]; Stefano D'Ugo [Department of Surgery, "V. Fazzi" Hospital, Lecce, Italy]; Stoica Bogdan [Carol Davila Medicine University]; Stylianos Kapiris [3rd Department of Surgery, Evangelismos Hospital, Athens, Greece]; Summi Karn [AIIMS, Rishikesh]; Sylvia Krivan [Attica General Hospital KAT]; Sztipits Tamás [National Institute of Oncology, Budapest, Hungary]; Tahsin [Mersin University]; Tevfik Kıvılcım Uprak; Thalia Petropoulou [Aretaieion University Hospital]; Theodoros Sidiropoulos [4th Department of Surgery, Attikon University Hospital, National and Kapodistrian University of Athens]; Tigabu Daniel Ayase [Hawassa University, Ethiopia]; Tijmen Koëter [TerGooi MC]; Tommaso Dominioni [Fondazione IRCCS Policlinico San Matteo - Pavia]; Tommaso Fontana [P.O. “Vittorio Emanuele” Gela]; Tommaso Violante [Alma Mater Studiorum Università di Bologna]; Usman Gwaram [Baze University Hospital, Abuja]; Uzochi Ebochue [University of Maiduguri Teaching Hospital]; Valentin Calu [University of Medicine and Pharmacy Carol Davila, Bucharest]; Venkatesh Munikrishnan [Apollo Hospitals, Chennai]; Vikas Sud [Stoke Mandeville Hospital]; Vincenzo Vigorita [University Complex Hospital of Vigo]; Vittoria Bellato [Minimally Invasive Surgery, Tor Vergata University of Rome]; Vittorio Bresadola [University of Udine]; Vusal Aliyev [Bogazici Academy for Clinical Sciences, İstanbul, Turkey]; Wasim MD [Manipal Hospital Jayanagar, Bangalore]; Zafar Ahmed Khan [University of the Witwatersrand]; Zafer Şenol [Istanbul Sultan Abdülhamid Han Research and Training Hospital]; Zainab Obaid Jaddoa; Zampitis Nikolaos [Tzaneio General Hospital of Piraeus]; Zhang Yankai [Peking University International Hospital]; Zoe Garoufalia [Cleveland Clinic Florida]; Zubair ud Din [Hayatabad Medical Complex, Peshawar, Khyber Pakhtunkhwa, Pakistan]; Zygomalas Apollon [Olympion General Clinic of Patras]; Εlissavet Anestiadou [Papanikolaou General Hospital of Thessaloniki]; Harish Neelamraju Lakshmi; Tomara Nefeli-Kaiti; Mehmet Ayhan KuU; Paola De Nardi; Iryivuze Olivier; Sudhir Dhaygude; Nadzlee Harith Bin Paisol; Sidra Rauf; Denis Tsepov; Mahmudul Hasan; Justin Alberts; Akshay Bavikatte Prasannakumar; Dorottya Turu; Ahmed Adam; Cumhur Yesildal; Hazim Eltyeb; Pablo Baeza-Ibáñez; Mohd. Azharuddhin; Ahmad Amhaimed; Claudia Alejandra Antón Velez; Ouazzani Et-Tayab; William Speake; Emre Furkan Kirkan; Megha Mishra; Ioannis Paraskevopoulos; Biagio Picardi; Braulio Francisco Reyes Méndez; Victor Bako; Riza Deryol; Henok Teshome Ayele.
